# Supplementary material for: A structured coalescent model reveals deep ancestral structure shared by all modern humans
Source: Nat Genet. 2025 Mar 18;57(4):856–64. doi: 10.1038/s41588-025-02117-1 (PMC11985351; doi:10.1038/s41588-025-02117-1)
Supplement: Supplementary file 1 — Supplementary Figs. 1–25, Supplementary Tables 1–7 and Supplementary Note. [file 41588_2025_2117_MOESM1_ESM.pdf]

nature genetics

Supplementary information https://doi.org/10.1038/s41588-025-02117-1

A structured coalescent model reveals deep ancestral structure shared by all modern humans

In the format provided by the authors and unedited

Contents

1 Supplementary Figures and Tables **2**

2 Supplementary Text **18**

2.1 Previous work . . . 18
2.2 PSMC's HMM and notation . . . 18
2.2.1 Emissions and transitions . . . 19
2.2.2 Discretisation . . . 21
2.2.3 Parameter inference . . . 21
2.3 *cobraa*'s HMM . . . 22
2.3.1 Transition probabilities under the structured model . . . 22
2.3.2 Demonstration of correctness . . . 33
2.4 Inference from *cobraa* on simulations . . . 37
2.4.1 Identifiability of structured model parameters . . . 38
2.5 *cobraa-path*'s HMM . . . 44
2.5.1 Demonstration of correctness . . . 45

1 Supplementary Figures and Tables

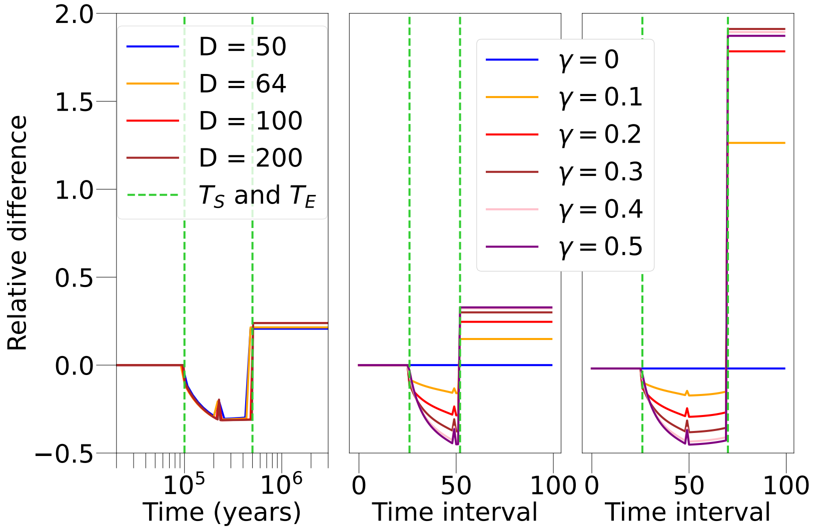

Supplementary Figure 1: Relative difference in transition matrices for different model parameters. For the matrix ξ in Main Fig. 2b, we plot values in the rows that correspond to a given coalescence time in the structured period. The x-axis corresponds to the time (left panel, time in years; middle and right panels, state indices of the HMM), with the vertical, green, dashed lines indicating the split and admixture time of the structured model. Left panel has the same *pulse* parameters but an increasing number of discrete time intervals, D. The relative difference does not increase or decreases as function of D, indicating that this result is not a consequence of time discretisation. Middle and right panels show how the relative difference increases as does the admixture fraction, γ. The right panel has a longer period of separation between populations A and B than does the left or middle panel, which also increases the relative difference.

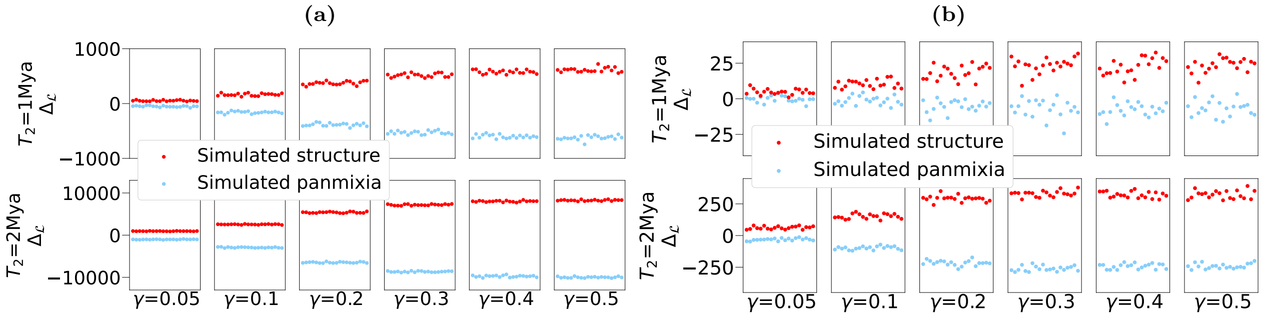

Supplementary Figure 2: Differences in log-likelihood ($\mathcal{L}$) between a simulated structured or unstructured model, each with the same coalescence rate. We simulated 20 replicates of each model, with a sequence length of 100Mb, population size $N = $ 2e+4, mutation rate per base pair per generation $\mu = $ 1.25e-8, and recombination rate per neighbouring base pairs per generation $r = $ 1e-8. When simulating from the structured model we let the admixture fraction vary between 5% and 50%, set T_1 as 200ka and T_2 as 1Ma or 2Ma; when simulating from the unstructured model we create a series of size changes such that the coalescence rate is exactly the same as the corresponding structured model. For each simulation, we calculate the $\mathcal{L}$ of the data under a structured and unstructured model. Thus there are 4 possibilities: structured simulation with structured inference, structured simulation with unstructured inference, unstructured simulation with structured inference, and unstructured simulation with unstructured inference. We plot the LL difference between the structured and unstructured inference, $\Delta_{\mathcal{L}} = \mathcal{L}_S - \mathcal{L}_U$, and colour the points red when the simulation is structured and blue when the simulated is unstructured. **(a)** Data are the sequence of simulated coalescence times across the genome (i.e. the probability of the Markov chain). We see clearly that the log-likelihood is able to distinguish the two histories, because $\Delta_{\mathcal{L}}$ is positive when the simulated evolutionary history is structured and negative when the simulated evolutionary history is panmictic. This is true even for a small admixture fraction, and the $\mathcal{L}$ differences increase as does the admixture fraction or the separation time. **(b)** Data are the sequence of simulated heterozygotes or homozygotes across the genome (i.e. the probability of the HMM). If both the admixture fraction and separation time are sufficiently large then the two evolutionary histories are distinguishable using their $\mathcal{L}$, though it seems small admixture fractions are not identifiable with a 100Mb sequence. The magnitude of difference in likelihoods between (a) and (b) reflect the uncertainty in inferring the coalescence time. The increase in $\Delta_{\mathcal{L}}$ as the admixture fraction or separation time increase is consistent with the relative error plots as seen in Supplementary Fig. 1.

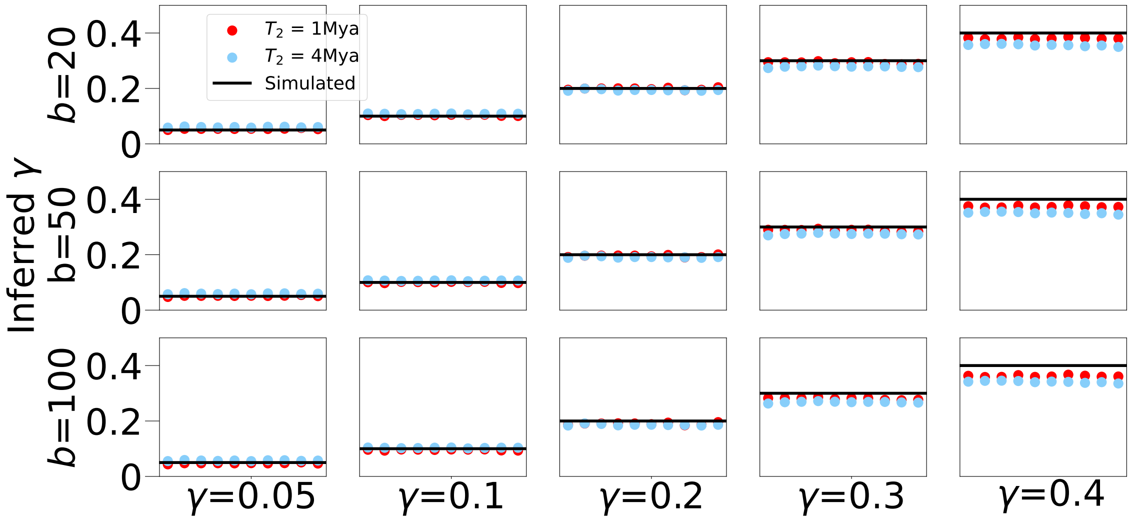

Supplementary Figure 3: Ability of *cobraa* to infer the admixture fraction γ, when the population sizes and split/admixture times are known, for various bin sizes b.

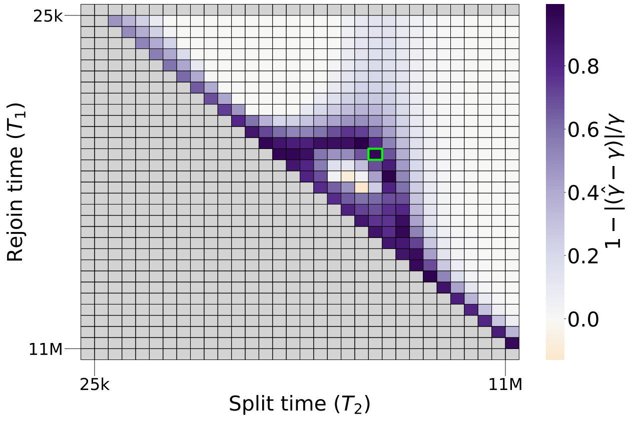

Supplementary Figure 4: The error between the *cobraa* inferred admixture fraction from each T_1, T_2 pairing and the simulated admixture fraction. The heatmap shows one minus the relative error between the inferred value $\hat{\gamma}$ and the simulated value γ. The corresponding log-likelihood for each pairing is shown in Main Fig. 3. The simulated T_1,T_2 pair is highlighted in the green cell.

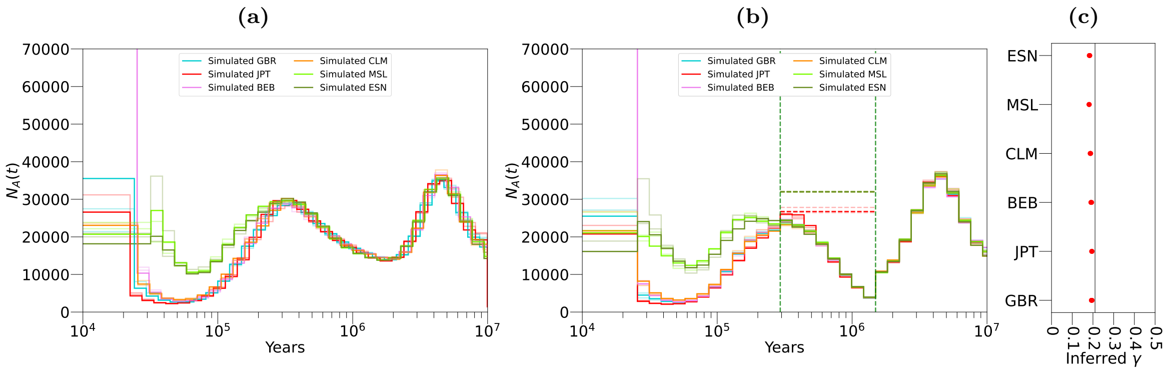

Supplementary Figure 5: We simulated 3 replicates of 2Gb of sequence data from the structured model as inferred by *cobraa* on real 1000GP data (Main Fig. 4b and 4c). **(a)** We ran PSMC on these simulations, and found that the inference looks extremely similar to the PSMC inference on real data (Main Fig. 4a), suggesting our structured model is compatible with previous coalescence-based estimations that assume no admixture. **(b)** and **(c)** We ran *cobraa* on these simulations, to check that we would have power to infer such a structured model if it really happened. Shown in (b) are the estimated sizes of $N_A(t)$ (solid lines) , $N_B(t)$ (horizontal, dashed lines), and the split and admixture time (vertical, dashed, green lines); the inferred admixture fraction is shown in (c) in the red circles, with the mean simulated value shown in the vertical, black line.

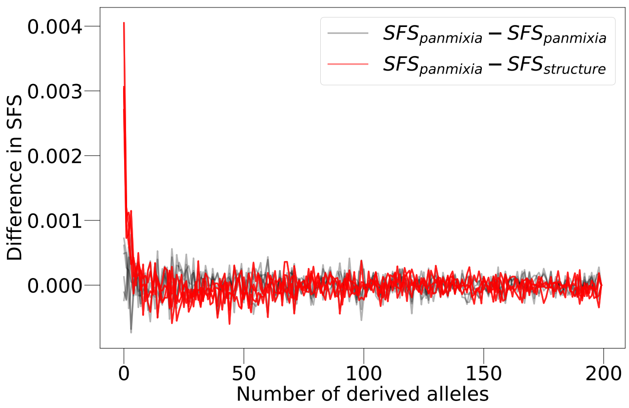

Supplementary Figure 6: With a panmictic and structured evolutionary history with the same coalescence rate, as shown in Main Fig. 2a, we simulated 200 individuals from each model and record the SFS. In red, we plot $SFS_{panmixia} - SFS_{structure}$, and see that rare alleles are more common under the panmictic model. In grey we show different replicates of $SFS_{panmixia} - SFS_{panmixia}$ to get an estimate of sampling noise. The differences in $SFS_{panmixia} - SFS_{structure}$ indicates that the SFS could in principle be used to overcome the pairwise coalescence rate identifiability problem as discussed by Mazet et al. [1, 2].

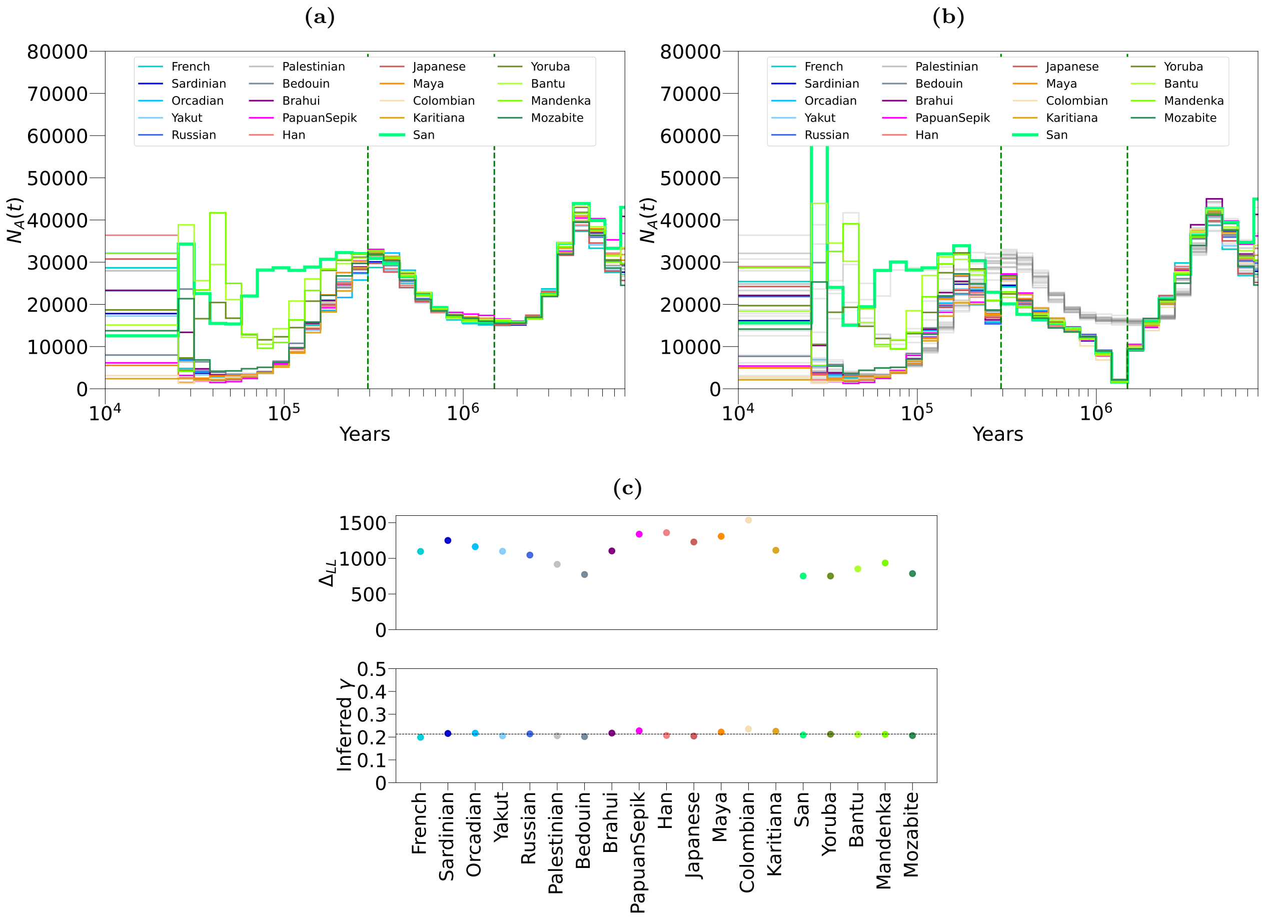

Supplementary Figure 7: Inference from PSMC and *cobraa* on 19 populations from the Human Genome Diversity Project, using one individual per population, iterating until convergence. **(a)** PSMC's estimate of $N_A(t)$. **(b)** *cobraa*'s estimate of $N_A(t)$, with the estimated split/admixture time shown in vertical, dashed, green lines. For direct comparison, the PSMC inference from (a) is also plotted in grey. **(c)** The top panel shows the difference between the log-likelihood from *cobraa*'s inference and PSMC's inference, $\Delta_{\mathcal{L}} = \mathcal{L}_S - \mathcal{L}_U$; the bottom panel shows *cobraa*'s inferred admixture fraction γ. The results on the HGDP dataset are extremely similar to those as inferred on the 1000GP data (Main Fig. 4).

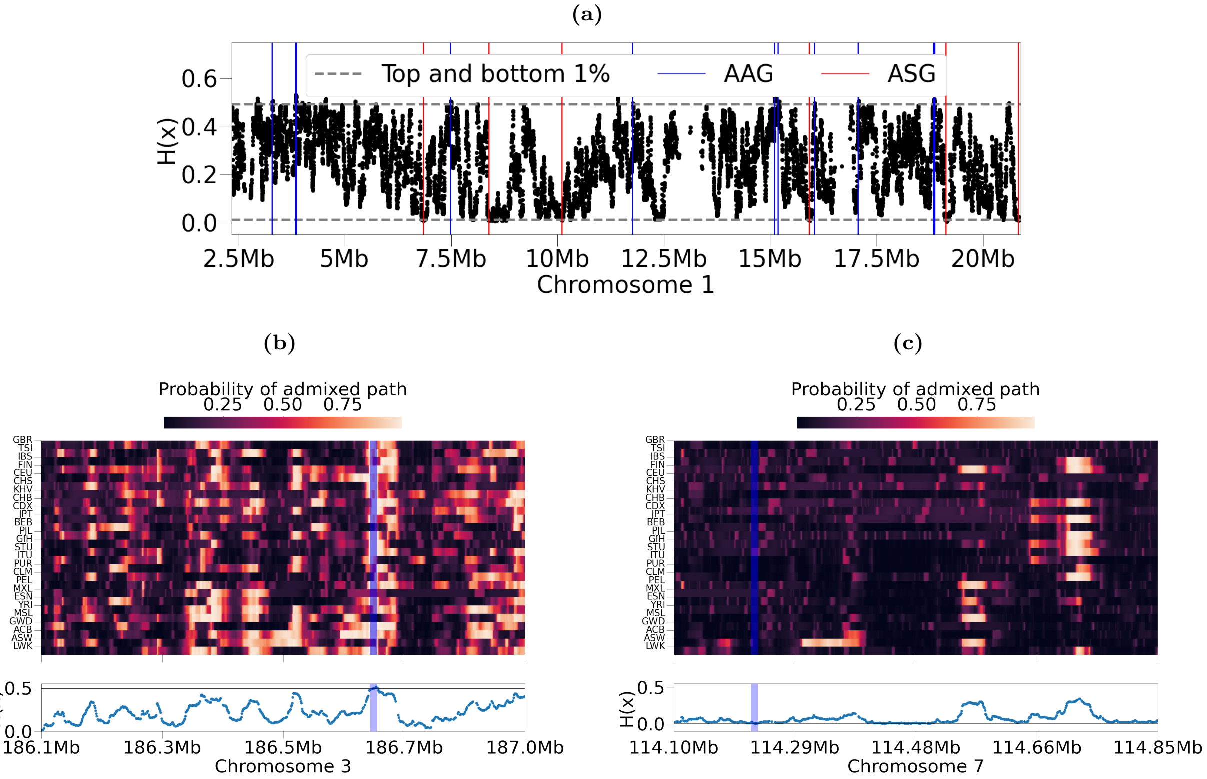

Supplementary Figure 8: *cobraa-path* can be used to infer the probability that one or both of the lineages went through the ghost population. Using these probabilities, we defined $H(x)$ to calculate the expected amount of admixture across all populations at each position. **(a)** An illustration of $H(x)$ across chromosome 1 (black dots), with the top and bottom 1% threshold indicated in the grey dashed line. Protein coding genes overlapping with the top or bottom 1% of $H(x)$ (AAGs and ASGs) are shown in blue and red, respectively, which illustrates that many regions exceeding the threshold values do not overlap with protein coding genes. Plotted values of $H(x)$ are shown with points, rather than a continuous line due to missing data causing discontinuity. **(b)** The top panel shows the probability of admixture for each population, on chromosome 3 around the gene *KNG1* (highlighted in blue), which is one of the 680 protein coding genes overlapping the regions in the top 1% of $H(x)$. The probability of admixture for population j is $P(c_x^j \in [AB, BB] | t_x^j > T_1, X^j)$, where x is the genomic position, t is the coalescence time at x, c is the ancestral lineage path at x, and X is all the observed data. The bottom panel shows the corresponding value of $H(x)$. **(c)** The top panel shows the probability of admixture for each population, on chromosome 7 around the gene *FOXP2* (highlighted in blue), which is one of the 1287 protein coding genes overlapping the regions in the bottom 1% of $H(x)$. The bottom panel shows the corresponding value of $H(x)$.

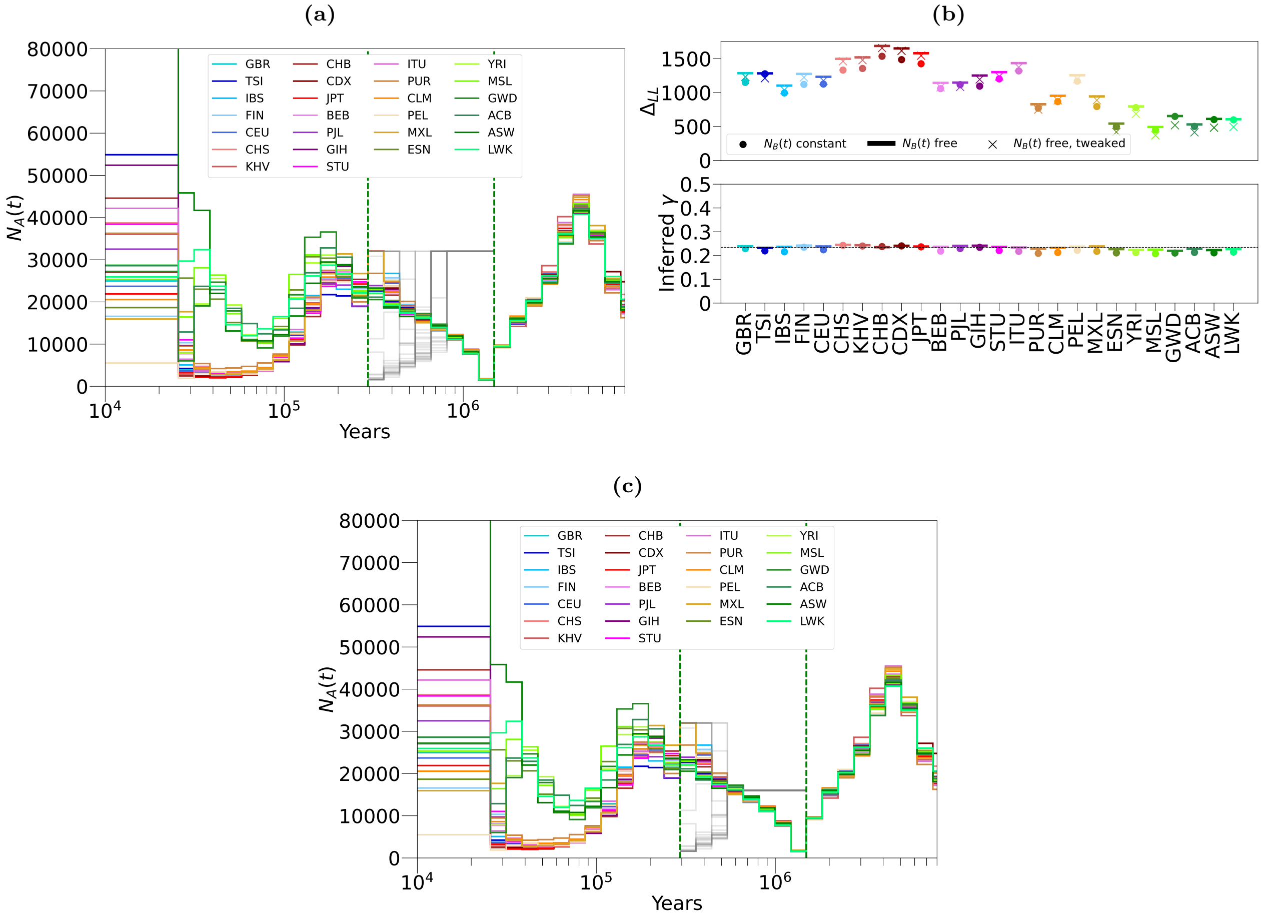

Supplementary Figure 9: Inference from *cobraa* on 26 populations from the 1000 Genomes Project, with $N_B(t)$ allowed to vary (contrary to Main Fig. 4 in which $N_B(t)$ was forced to be constant). **(a)** Estimates of $N_A(t)$ (coloured lines) and $N_B(t)$ (grey lines). **(b)** The difference in log-likelihood from *cobraa*'s and PSMC's inference, $\Delta_{\mathcal{L}} = \mathcal{L}_S - \mathcal{L}_U$ (top panel), and the inferred admixture fraction (bottom panel). The circles indicate $\Delta_{\mathcal{L}}$ and the inferred γ from the the model where $N_B(t)$ was enforced to be constant (i.e. are exactly the same as Main Fig. 4c), the horizontal lines indicate the inference with $N_B(t)$ allowed to vary, and the cross indicates the log-likelihood of the free $N_B(t)$ model after the size of $N_B(t)$ is reduced post divergence, as depicted in **(c)**.

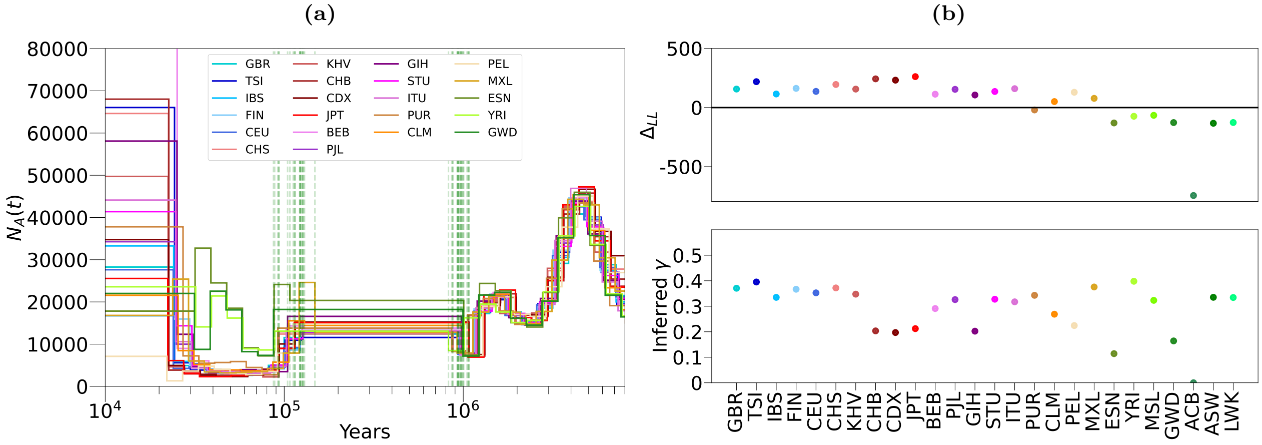

Supplementary Figure 10: Inference from *cobraa* on the 1000GP populations, when we enforce that $N_A(t)$ must be constant in the structured period. **(a)** Inference of $N_A(t)$ and the split/admixture times, **(b)** Estimates of the admixture fraction (top) and the difference in log-likelihood between *cobraa* and PSMC (bottom). We conclude a structured model with this constraint is not well supported by the data, as $\Delta_{\mathcal{L}}$ is often negative and the estimates of the admixture fraction have high variance.

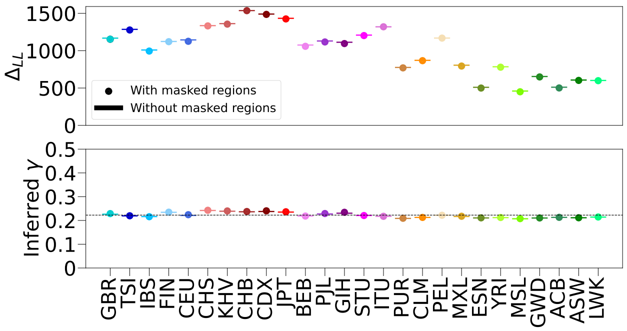

Supplementary Figure 11: Missing data in the HMM does not make a difference for inference. We reran PSMC and *cobraa* analysis after removing missing data from the observations (see Methods), and found the results to be indistinguishable. The circles indicate $\Delta_{\mathcal{L}}$ and the inferred γ from the full data as (i.e. are exactly the same as Main Fig. 4c), and the horizontal lines indicate the analysis after the missing data was removed.

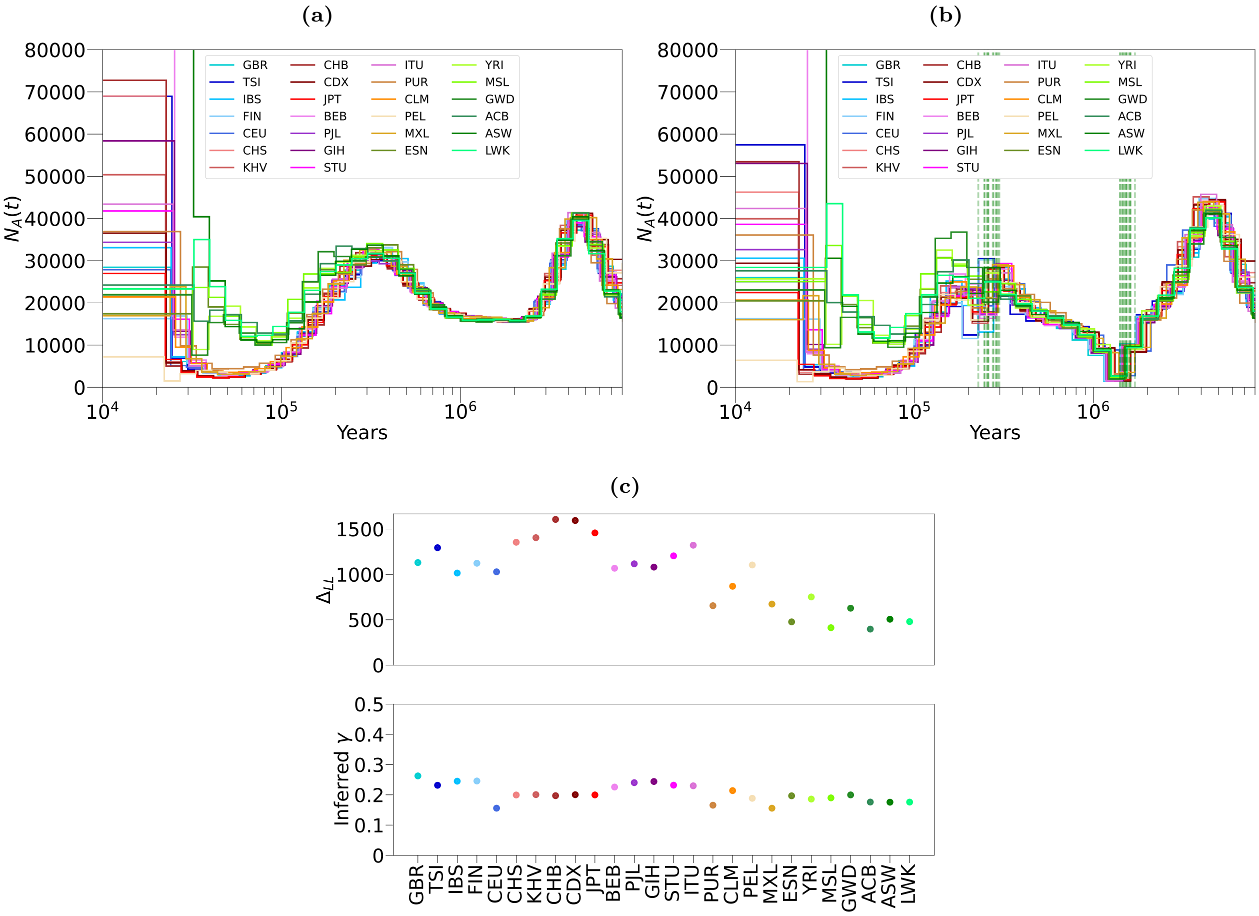

Supplementary Figure 12: Inference from PSMC and *cobraa* on 26 populations from the 1000 Genomes project, using one individual per population, with θ inferred from the data. **(a)** PSMC's estimate of $N_A(t)$. **(b)** *cobraa*'s estimate of $N_A(t)$, with the estimated split/admixture time shown in vertical, dashed, green lines. **(c)** The top panel shows the difference between the log-likelihood from *cobraa*'s inference and PSMC's inference, $\Delta_{\mathcal{L}} = \mathcal{L}_S - \mathcal{L}_U$; the bottom panel shows *cobraa*'s inferred admixture fraction γ.

(a)

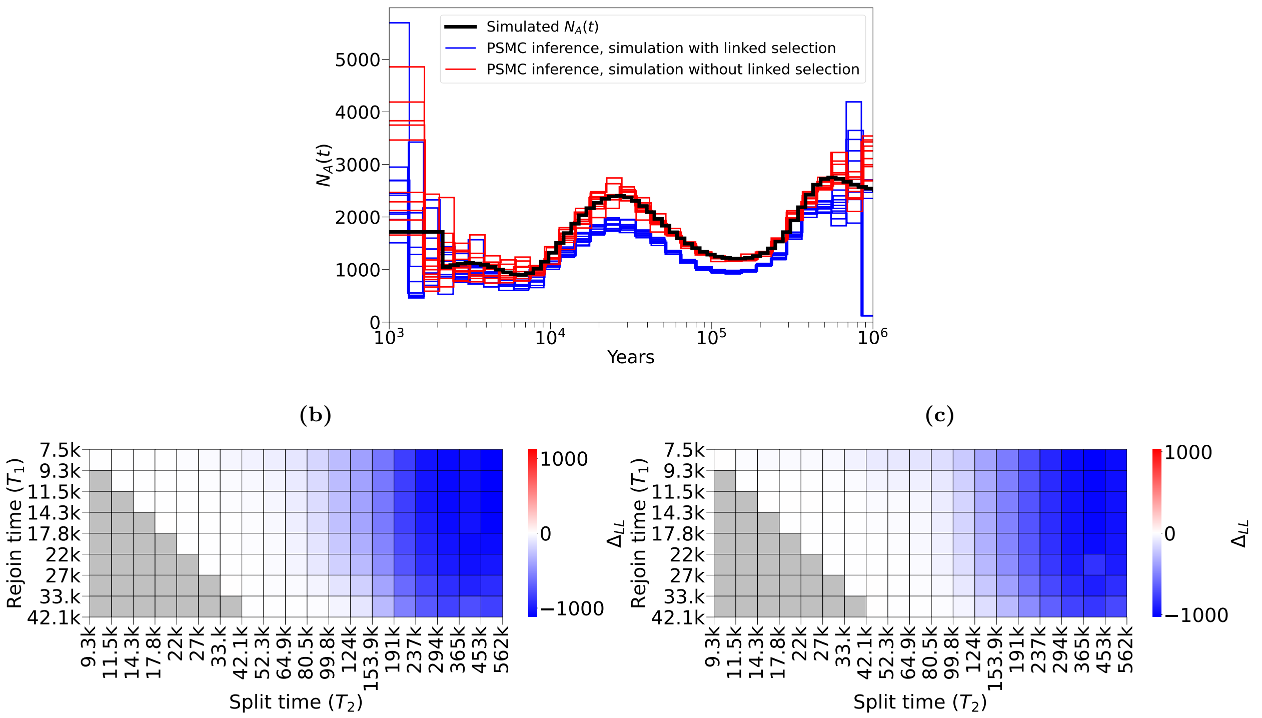

Supplementary Figure 13: Inference from PSMC and *cobraa* on simulations with and without widespread linked selection, taken from [3] (see Methods). **(a)** PSMC inference on a simulation with and without widespread linked selection. We ran *cobraa* over various pairings of T_1 and T_2, and calculated the log-likelihood difference between this and the inference from PSMC, $\Delta_{\mathcal{L}} = \mathcal{L}_S(\hat{T}_1, \hat{T}_2) - \mathcal{L}_U(\hat{T}_1, \hat{T}_2)$. In **(b)** and **(c)**, we show $\Delta_{\mathcal{L}}$ for a neutral simulation and simulation with widespread linked selection, respectively. The log-likelihood differences indicate that *cobraa* does not falsely infer linked selection as structure.

Population	Sample	# SNPs	# Masked bases	# Called bases	Heterozygosity
GBR	HG00118	1617906	721916763	2150511886	0.00075
TSI	NA20752	1637719	722969288	2149265625	0.00076
IBS	HG01783	1614003	731281619	2140269189	0.00075
FIN	HG00266	1619493	729407481	2142893467	0.00075
CEU	NA12718	1611666	721749959	2150584901	0.00074
CHS	HG00443	1518102	720272359	2152069281	0.0007
KHV	HG02113	1508267	723553886	2148789845	0.0007
CHB	NA18530	1521984	720811264	2151278579	0.0007
CDX	HG02373	1522518	716656317	2155682074	0.0007
JPT	NA18939	1503662	721812512	2150488705	0.00069
BEB	HG03006	1698194	717857271	2154406877	0.00078
PJL	HG03234	1681759	719513151	2152802272	0.00078
GIH	NA20845	1686133	722230248	2143732963	0.00078
STU	HG03753	1667039	728686017	2143519959	0.00077
ITU	HG03977	1657672	728197863	2144214469	0.00077
PUR	HG01171	1829263	720624146	2151746582	0.00085
CLM	HG01250	1719514	717744633	2154605688	0.00079
PEL	HG02285	1482859	723765263	2148505378	0.00069
MXL	NA19648	1602318	719980307	2152287788	0.00074
ESN	HG03515	2133476	723310219	2149247137	0.00099
YRI	NA18488	2144349	727775665	2144733225	0.00099
MSL	HG03212	2147856	724459558	2148121619	0.00099
GWD	HG02568	2104590	733799722	2138584844	0.00098
ACB	HG01882	2171991	725229153	2147260898	0.00101
ASW	NA19625	2156788	729430494	2143090371	0.001
LWK	NA19017	2175804	720231319	2152220886	0.00101

Supplementary Table 1: Sequence information for each sample we used in the 1000 Genomes Project. The third column is the number of SNPs that passed all filters. The fourth column is the number of bases that were marked as “missing data”. The fifth column is number of bases that were either homozygous or heterozygous that passed all filters. The sixth column is the average heterozygosity, calculated by dividing the number of heterozygous positions (second column) divided by the number of called bases (fourth column).

Population	dcCDS vs $Pr(c = k \vert X, t > T_1)$ $k = AA$	$k = BB$	$k = AB$	B-value vs $Pr(c = k \vert X, t > T_1)$ $k = AA$	$k = BB$	$k = AB$
GBR	-0.08	0.07	0.07	-0.27	0.27	0.20
TSI	-0.08	0.08	0.07	-0.28	0.33	0.22
IBS	-0.09	0.09	0.07	-0.27	0.32	0.21
FIN	-0.09	0.04	0.07	-0.29	0.23	0.20
CEU	-0.09	0.07	0.07	-0.28	0.32	0.21
CHS	-0.07	0.04	0.05	-0.27	0.18	0.19
KHV	-0.08	0.03	0.06	-0.28	0.18	0.20
CHB	-0.08	0.01	0.06	-0.28	0.15	0.19
CDX	-0.07	0.02	0.05	-0.28	0.15	0.19
JPT	-0.06	0.03	0.05	-0.26	0.17	0.18
BEB	-0.09	0.08	0.07	-0.28	0.33	0.22
PJL	-0.08	0.07	0.06	-0.28	0.32	0.21
GIH	-0.09	0.05	0.07	-0.29	0.23	0.21
STU	-0.08	0.08	0.07	-0.28	0.33	0.22
ITU	-0.08	0.07	0.07	-0.26	0.32	0.20
PUR	-0.1	0.09	0.08	-0.29	0.34	0.24
CLM	-0.08	0.08	0.07	-0.28	0.33	0.21
PEL	-0.06	0.06	0.06	-0.26	0.31	0.20
MXL	-0.08	0.07	0.07	-0.27	0.31	0.20
ESN	-0.1	0.09	0.09	-0.33	0.37	0.28
YRI	-0.11	0.10	0.10	-0.32	0.37	0.27
MSL	-0.11	0.10	0.10	-0.33	0.37	0.28
GWD	-0.11	0.10	0.09	-0.32	0.37	0.27
ACB	-0.11	0.10	0.10	-0.33	0.38	0.29
ASW	-0.11	0.10	0.09	-0.32	0.37	0.27
LWK	-0.11	0.10	0.10	-0.32	0.38	0.27
mean	-0.09	0.07	0.07	-0.29	0.30	0.23

Supplementary Table 2: Spearman correlation between the inferred probability of an admixed lineage path, $Pr(c = k|X, t > T_1)$, and the distance to coding sequence (dcCDS) or B-value, for each population on all chromosomes.

Biological function	# Human genes	# AAGs	Expected	Enrichment	p-value	FDR
Neuron cell-cell adhesion	16	6	0.52	11.63	4.42e-5	4.21e-2
Startle response	30	8	0.97	8.27	1.85e-5	3.53e-2
Neuron recognition	45	9	1.45	6.20	4.06e-5	4.12e-2
Neurotransmitter transport	142	17	4.58	3.71	1.04e-5	2.27e-2
Extracellular matrix organization	278	24	8.96	2.68	3.00e-5	4.16e-2
Chemical synaptic transmission	420	34	13.54	2.51	3.12e-6	1.19e-2
Circulatory system process	508	36	16.38	2.20	2.58e-5	3.93e-2
Regulation of biological quality	2849	132	91.87	1.44	2.48e-5	4.20e-2
Gene expression	2491	44	80.32	0.55	6.04e-6	1.53e-2

Supplementary Table 3: Biological processes associated with the 680 admixture abundant genes (AAGs). First column is the reported biological process reported from Panther's [4] analysis, second column is number of human genes associated with this process (out of 20,592), third column is number of AAGs associated with this process, fourth column is expected number of genes assuming a random sampling, fifth column is the enrichment with respect to the number of expected genes, sixth column is the two-sided p-value from Fisher's Exact Test, and the seventh column is the Benjamini-Hochberg False Discovery Rate. Full information including parent processes are available for download online.

Biological function	# Human genes	# ASGs	Expected	Enrichment	p-value	FDR
Pre-miRNA processing	14	7	0.85	8.28	1.10e-4	1.11e-2
Cortical actin cytoskeleton organization	41	10	2.48	4.04	5.22e-4	4.12e-2
Golgi to plasma membrane transport	50	11	3.02	3.64	5.91e-4	4.62e-2
Regulation of intracellular steroid hormone receptor signaling pathway	74	15	4.47	3.36	1.42e-4	1.37e-2
Homophilic cell adhesion via plasma membrane adhesion molecules	167	33	10.09	3.27	3.37e-8	9.02e-6
Regulation of embryonic development	92	16	5.56	2.88	4.00e-4	3.33e-2
Protein deubiquitination	109	18	6.58	2.73	3.13e-4	2.73e-2
Peptidyl-serine phosphorylation	163	26	9.85	2.64	4.23e-5	5.16e-3
Cytosolic transport	133	21	8.03	2.61	2.95e-4	2.58e-2
Protein dephosphorylation	137	21	8.28	2.54	3.48e-4	2.97e-2
Mesenchymal cell differentiation	172	24	10.39	2.31	4.66e-4	3.80e-2
Vesicle localization	181	25	10.93	2.29	3.70e-4	3.10e-2
Proteasome-mediated ubiquitin-dependent protein catabolic process	297	41	17.94	2.29	4.72e-6	8.00e-4
Endosomal transport	265	34	16.01	2.12	1.26e-4	1.23e-2
Chromatin remodeling	621	79	37.52	2.11	6.13e-9	1.95e-6
Regulation of GTPase activity	298	37	18.00	2.06	1.15e-4	1.15e-2
Cell division	525	60	31.72	1.89	1.12e-5	1.65e-3
Regulation of neuron projection development	459	52	27.73	1.88	6.14e-5	6.89e-3
Regulation of neuron projection development	459	52	27.73	1.88	6.14e-5	6.89e-3
Positive regulation of cell projection organization	358	40	21.63	1.85	6.18e-4	4.73e-2
DNA repair	519	57	31.35	1.82	5.16e-5	6.05e-3
Protein ubiquitination	625	67	37.76	1.77	2.59e-5	3.46e-3
Positive regulation of catabolic process	523	56	31.60	1.77	1.17e-4	1.16e-2
Regulation of cell cycle phase transition	434	46	26.22	1.75	6.31e-4	4.81e-2
Neuron projection morphogenesis	491	51	29.66	1.72	4.64e-4	3.80e-2
MRNA metabolic process	622	64	37.58	1.70	1.05e-4	1.07e-2
DNA-templated transcription	539	55	32.56	1.69	4.20e-4	3.46e-2
Cell cycle process	880	89	53.16	1.67	9.52e-6	1.44e-3
Brain development	742	75	44.83	1.67	4.17e-5	5.21e-3
Heart development	566	57	34.19	1.67	4.17e-4	3.45e-2
Mitotic cell cycle	594	59	35.88	1.64	5.54e-4	4.35e-2
Microtubule-based process	824	81	49.78	1.63	5.15e-5	6.08e-3
Regulation of anatomical structure morphogenesis	845	82	51.05	1.61	8.33e-5	8.58e-3
Protein localization to organelle	739	71	44.64	1.59	3.50e-4	2.97e-2
Protein transport	1090	103	65.85	1.56	2.09e-5	2.84e-3
Positive regulation of cell differentiation	871	82	52.62	1.56	1.89e-4	1.76e-2
Regulation of cellular component biogenesis	974	89	58.84	1.51	2.39e-4	2.16e-2
Intracellular signal transduction	1514	136	91.46	1.49	1.17e-5	1.71e-3
Negative regulation of DNA-templated transcription	1279	112	77.27	1.45	2.00e-4	1.84e-2
Regulation of protein modification process	1230	106	74.31	1.43	4.98e-4	4.00e-2
Positive regulation of DNA-templated transcription	1711	147	103.36	1.42	4.22e-5	5.19e-3
Regulation of intracellular signal transduction	1781	151	107.59	1.40	5.78e-5	6.63e-3
Negative regulation of response to stimulus	1664	140	100.53	1.39	1.60e-4	1.52e-2
Positive regulation of signal transduction	1568	130	94.73	1.37	4.73e-4	3.84e-2
Cellular component assembly	2459	196	148.55	1.32	1.18e-4	1.16e-2
G protein-coupled receptor signaling pathway	1248	39	75.39	0.52	5.44e-6	8.92e-4
Adaptive immune response	681	11	41.14	0.27	6.50e-8	1.60e-5
Lymphocyte mediated immunity	257	2	15.53	0.13	6.48e-5	7.00e-3
Detection of chemical stimulus involved in sensory perception of smell	443	1	26.76	0.04	1.63e-10	9.54e-8
Antimicrobial humoral response	150	0	9.06	< 0.01	2.69e-4	2.39e-2

Supplementary Table 4: Biological processes associated with the 1287 admixture scarce genes (ASGs). First column is the reported biological process reported from Panther's [4] analysis, second column is number of human genes associated with this process (out of 20,592), third column is number of ASGs associated with this process, fourth column is expected number of genes assuming a random sampling, fifth column is the enrichment with respect to the number of expected genes, sixth column is the two-sided p-value from Fisher's Exact Test, and the seventh column is the Benjamini-Hochberg False Discovery Rate. Full information including parent processes are available for download online.

ID	Population
ASW	African Ancestry in Southwestern USA
ACB	African Caribbeanin Barbados
BEB	Bengali in Bangladesh
GBR	British from England and Scotland
CDX	Chinese Dai in Xishuangbanna, China
CLM	Colombian in Medellin, Colombia
ESN	Esan in Nigeria
FIN	Finnish in Finland
GWD	Gambian in Western Division - Mandinka
GIH	Gujarati Indians in Houston, Texas, United States
CHB	Han Chinese in Beijing, China
CHS	Han Chinese South, China
IBS	Iberian populations in Spain
ITU	Indian Telugu in the UK
JPT	Japanese in Tokyo, Japan
KHV	Kinh in Ho Chi Minh City, Vietnam
LWK	Kenya Luhya in Webuye, Kenya
MSL	Sierra Leone Mende in Sierra Leone
MXL	Mexican Ancestry in Los Angeles, California, United States
PEL	Peruvian in Lima, Peru
PUR	Puerto Rico Puerto Rican in Puerto Rico
PJL	Punjabi in Lahore, Pakistan
STU	Sri Lankan Tamil in the UK
TSI	Toscani in Italy
YRI	Yoruba in Ibadan, Nigeria
CEU	Utah residents with Northern and Western European ancestry from the CEPH collection

Supplementary Table 5: Triplet codes used for each of the 26 populations in the 1000 Genomes Project.

Population, sample	AIC_U	AIC_S	AIC_U-AIC_S
GBR, HG00118	11446280	11443982	2298
TSI, NA20752	11592765	11590218	2547
IBS, HG01783	11462286	11460304	1982
FIN, HG00266	11481262	11479029	2233
CEU, NA12718	11427413	11425170	2243
CHS, HG00443	10785904	10783249	2655
KHV, HG02113	10741543	10738840	2703
CHB, NA18530	10819428	10816364	3064
CDX, HG02373	10814788	10811825	2963
JPT, NA18939	10705411	10702569	2842
BEB, HG03006	11944455	11942346	2109
PJL, HG03234	11833265	11831039	2226
GIH, NA20845	11892662	11890479	2183
STU, HG03753	11799049	11796653	2396
ITU, HG03977	11748314	11745683	2631
PUR, HG01171	12793701	12792170	1531
CLM, HG01250	12067152	12065427	1725
PEL, HG02285	10550718	10548389	2329
MXL, NA19648	11299068	11297487	1581
ESN, HG03515	14722313	14721322	991
YRI, NA18488	14832185	14830635	1550
MSL, HG03212	14817779	14816888	891
GWD, HG02568	14631867	14630578	1289
ACB, HG01882	14976777	14975783	994
ASW, NA19625	14932119	14930923	1196
LWK, NA19017	14972468	14971279	1189

Supplementary Table 6: For the inferred structured and unstructured model as shown in Main Fig. 4, we calculate the AIC. Even though the structured model has more parameters (37) than the unstructured model (33), the AIC is substantially lower for the structured model which suggests the better fit is not due to it having more parameters.

	Training		Testing	
Population	Sample	$\Delta_{\mathcal{L}}$	Sample	$\Delta_{\mathcal{L}}$
GBR	HG00118	1153.3	HG00119	1140.1
TSI	NA20752	1277.0	NA20753	1101.0
IBS	HG01783	994.9	HG02221	882.7
FIN	HG00266	1120.5	HG00267	963.9
CEU	NA12718	1125.6	NA12748	1133.9
CHS	HG00443	1331.8	HG00445	1412.0
KHV	HG02113	1355.6	HG02141	1368.4
CHB	NA18530	1535.8	NA18534	1320.4
CDX	HG02373	1485.8	HG02394	1494.4
JPT	NA18939	1425.2	NA18941	1295.6
BEB	HG03006	1058.4	HG03007	1276.4
PJL	HG03234	1116.8	HG03235	1083.2
GIH	NA20845	1095.4	NA20847	1124.5
STU	HG03753	1202.3	HG03760	1132.7
ITU	HG03977	1319.8	HG03780	1263.1
PUR	HG01171	769.8	HG01173	872.5
CLM	HG01250	866.2	HG01251	994.4
PEL	HG02285	1168.4	HG02286	1315.7
MXL	NA19648	794.1	NA19649	908.3
ESN	HG03515	499.5	HG03514	647.0
YRI	NA18488	778.9	NA19222	757.4
MSL	HG03212	449.7	HG03055	694.6
GWD	HG02568	648.5	HG02759	391.9
ACB	HG01882	501.0	HG01883	526.8
ASW	NA19625	601.9	NA19700	600.7
LWK	NA19017	598.4	NA19019	609.6

Supplementary Table 7: Using the inferred parameters on the seen data (training), we calculate the log-likelihood of unseen data (testing) for a structured and unstructured model. The strongly positive $\Delta_{\mathcal{L}} = \mathcal{L}_{\mathcal{S}} - \mathcal{L}_{\mathcal{U}}$ in the test data indicates the better fitting structured model is not due to overfitting.

2 Supplementary Text

2.1 Previous work

The sequentially Markovian coalescent (SMC) [5] was introduced to describe analytically the probability of local changes in ancestry. Moving left to right across a genome sequence, it describes the probability of the next tree height (i.e. coalescence time) as conditionally dependent only on the current tree height, as opposed to the whole genealogy of the sequence thus far [6], enabling tractable inference. Marjoram and Wall proposed a modification to the SMC model [7], which more accurately models the coalescent with recombination [8] (previously, authors have used SMC' to denote the modification proposed by Marjoram and Wall, though here and in the main text we use SMC to mean the modified version). By assuming panmixia and using a discretised version of the conditional distribution from SMC in the transition matrix of its hidden Markov model (HMM), the pairwise sequentially Markovian coalescent (PSMC) [9] infers the changes in the effective population size over time and the coalescence times across the genome.

Wang et al. [10] introduced MSMC-IM to estimate a time-dependent rate of gene flow between two sampled populations. Given sequences from a pair of populations, it uses MSMC2 to infer coalescence rates within and across these populations, then fits a continuous Isolation-Migration model to these. It assumes that the migration rate was symmetric, and does not attempt to interpret coalescence rates in terms of population size. Simulations from Shchur et al. [11] indicated that PSMC's transition matrix has information that may be able to distinguish structure from panmixia. They developed MiSTI, which uses the joint SFS from two diploid sequences to estimate time-dependent size changes and continuous, non-symmetric migration rates.

2.2 PSMC's HMM and notation

In this section we review the model underlying PSMC, and introduce the notation that we use throughout. The original PSMC [9] uses Cardin and McVean's SMC model [5], which is sufficient for accurate parameter inference, but MSMC, MSMC2, and PSMC+ [12, 10, 3] use Marjoram and Wall's SMC model [7], which is a more accurate approximation of the coalescent with recombination [8]. For simplicity, henceforth we use "PSMC" to refer to a non-implementation specific version of PSMC using the Marjoram and Wall's SMC framework.

We measure time, t, in coalescent units going backwards in time (i.e. $t = 0$ is the present). Taking two lineages from population A at $t = 0$, PSMC infers the coalescence rate $\lambda_A(t)$ and uses

$$N_A(t) = \frac{1}{2\lambda_A(t)} \tag{1}$$

to estimate the changes through time in the effective size of population A.

2.2.1 Emissions and transitions

PSMC is a HMM where the observations are the sequence of homozygotes or heterozygotes across the genome, which we label $X = (x_1, x_2, \ldots, x_L)$ where L is the length of the sequence and $x_i = 0$ for a homozygote, $x_i = 1$ for a heterozygote and $x_i = -1$ for missing data. The latent variable at each position is the coalescence time, or time to most recent common ancestor (TMRCA), which we denote $Z = (z_1, z_2, ..., z_L)$, where theoretically z_i takes values $[0, \infty)$. We make the infinite sites assumption that at most one mutation can occur at any site, therefore at a single base pair level the emission probabilities are:

$$\begin{aligned} P(x = 0|z = t) &= e^{-\theta t} \\ P(x = 1|z = t) &= 1 - e^{-\theta t} \\ P(x = -1|z = t) &= 1 \end{aligned} \tag{2}$$

where $\theta = 4N_A\mu$ is the scaled mutation rate, N_A is the diploid, long-term effective population size, μ is the per base per generation mutation rate, and t is measured in coalescent units. By chopping the genome sequence into bins of b base pairs, we can achieve a linear computational speed-up by a factor of b. In each bin there are then h heterozygotes, m missing bases and $b - m - h$ homozygotes, with $0 \leq m, h \leq b$. The number of heterozygotes in a bin can then be modelled as a Poisson, and so the emission probabilities are thus:

$$P(x = (h, m)|z = t, b) = \frac{((b - m)(\theta t))^h e^{-(b-m)\theta t}}{h!} \tag{3}$$

Denote the i'th locus as having TMRCA s and the $(i + 1)$'th locus having TMRCA t. Let R and R' denote the instance or lack of a recombination, respectively, between loci i and $i + 1$. The probability of no recombination is $P(R'|z_i = s) = e^{-\rho s}$, where $\rho = 4N_A r$ is the scaled recombination rate, and r is recombination rate per neighbouring base pairs per generation. Because under the SMC recombination can occur at most once we take $P(R|z_i = s) = 1 - P(R'|z_i = s) = 1 - e^{-\rho s}$. With the sequence in bins of size b, the probability of not recombining between neighbouring bins is $P(R'|z_i = s, b) = e^{-\rho sb}$ so the probability of at least one recombination occurring is $P(R|z_i = s, b) = 1 - e^{-\rho sb}$. In what follows, for simplicity we typically do not write the b term explicitly.

The probability of transitioning between $z_i = s$ and $z_{i+1} = t$ is governed by the SMC model [7]. For brevity let $P(t|s) = P(z_{i+1} = t|z_i = s)$ denote the probability of transitioning under the SMC. Considering the presence or absence of recombination, we can write this as

$$P(t|s) = P(t, R'|s) + P(t, R|s) \tag{4}$$

but $P(t, R'|s) = 0$ if $t \neq s$ and $P(t, R|s) = P(t|R, s)P(R|s)$ so we have

$$P(t|s) = P(t, R'|s) + P(R|s)P(t|R, s). \tag{5}$$

Using the SMC model, we use the word "floating" to describe a lineage that has undergone recombination but is yet uncoalesced, and we use "solid" to denote the remaining unrecombined lineages. Given that a recombination has occurred there are three distinct types of coalescent event:

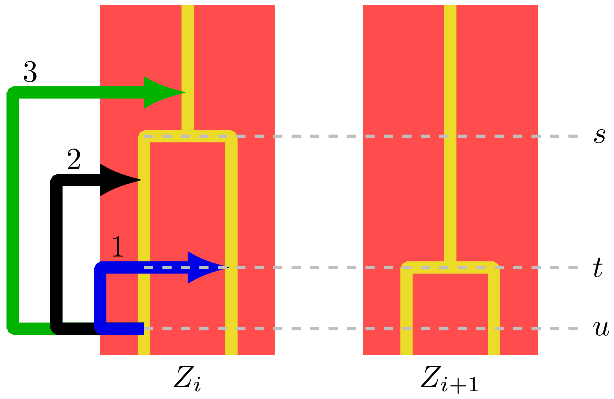

Supplementary Figure 14: A depiction of the SMC framework for locus i and $i+1$. The tree height (i.e. TMRCA) at locus i is s and at $i+1$ is t. Under the SMC model, given that a recombination has occurred at time u there are three possible types of coalescence event. The coloured arrows depict the "floating" lineage, which can either coalesce to the branch on which recombination did not occur (1), the branch on which recombination did occur (2), or the ancestral branch (3). In the diagram shown, a type 1 event is shown.

1. The floating lineage coalesces to the other solid lineage at time $t < s$
2. The floating lineage coalesces back to the same branch on which the recombination occurred, resulting in $t = s$
3. The floating lineage coalesces to the solid ancestral lineage at time $t > s$

A depiction of this process is given in Supplementary Fig. 14. We note that Cardin and McVean's SMC model only allows events 1 or 3.

Let u denote the time at which a recombination occurred (then strictly $u \leq t$ and $u < s$). If a type 1 event occurs, we must have no coalescence with either solid lineages between u and t, then a coalescence at time t to the branch that did not recombine. For a type 2 event, we must have the floating lineage coalesce with the lineage on which recombinaton occurred, at any time between u and s. For a type 3 event, we must have no coalescence between u and t on either solid branches, and subsequently no coalescence between s and t on the single ancestral solid lineage. Define

$$L_A(t_1, t_2) = e^{-\int_{t_1}^{t_2} \lambda_A(x)dx} \tag{6}$$

then we can write the probability of transitioning under the SMC' as:

$$\begin{aligned} P(t|s) = \delta(t-s)\left(P(R'|s) + P(R|s)\frac{1}{2s}\int_0^t (1 - L(u,t))^2 du\right) + \\ P(R|s)\frac{1}{s}\begin{cases} \int_0^t \lambda(t)L(u,t)^2 du & \text{if } t < s \\ \int_0^s \lambda(t)L(u,s)^2 L(s,t)du & \text{if } t > s \end{cases} \end{aligned} \tag{7}$$

Where we integrate over u because it is a variable with values in $[0, min(s,t)]$, the $1/s$ terms are a normalisation constant for the integration over u, and δ is the Kronecker delta which handles presence/absence

of recombination. See [10] for the full derivation.

2.2.2 Discretisation

Primarily the goal of PSMC is to infer changes in the effective population size over time from some population A. To do this, PSMC discretises the space of TMRCAs into D time intervals by partitioning $[0, \infty)$ into a state space of D time intervals with boundaries $\tau = (\tau_0, \ldots, \tau_D)$ which for $i \in (1, \ldots, D-1)$ are defined by

$$\tau_i = \omega exp\left(\frac{i}{D}log\left(1 + \frac{T_{max}}{\omega}\right) - 1\right) \tag{8}$$

where ω and T_{max} control their spread, and $\tau_0 = 0$ and $\tau_D = \infty$. The set of hidden states in the HMM is $T = \{T_1, ..., T_D\}$ and if the TMRCA at locus i is t with $\tau_\alpha \leq t < \tau_{\alpha+1}$ then $z_i = T_\alpha$. Denote the set of D inverse effective population size parameters with $\lambda_A = (\lambda_{A_1}, \ldots, \lambda_{A_D})$. Suppose that t is contained within interval α, and s in interval β, i.e. $\tau_\alpha \leq t < \tau_{\alpha+1}$ and $\tau_\beta \leq s < \tau_{\beta+1}$. To get the discrete emission probabilities, in MSMC2 [12] Malaspinas et al. integrate between the neighbouring boundaries combined with the stationary distribution $\pi(t)$, though we simply take the midpoint:

$$\begin{aligned} P(x = (h, m)|z = T_\alpha, b) &= P\left(x = (h, m)|z = (\tau_\alpha + \tau_{\alpha+1})/2, b\right) \\ &= \frac{((b-m)(\theta(\tau_\alpha + \tau_{\alpha+1})/2))^h e^{-(b-m)\theta(\tau_\alpha+\tau_{\alpha+1})/2}}{h!} \end{aligned} \tag{9}$$

Similarly, to create the transition matrix $Q(\alpha|\beta) = P(T_\alpha|\tau_\beta)$, Malaspinas et al. take the expected coalescence time in interval β:

$$\langle t\beta \rangle = \frac{1}{\pi(\beta)} \int_{\tau_\beta}^{\tau_{\beta+1}} t\pi(\beta)dt \tag{10}$$

and integrate out α:

$$Q(\alpha|\beta) = \int_{\tau_\alpha}^{\tau_{\alpha+1}} P(t|\langle t\beta \rangle)dt \tag{11}$$

2.2.3 Parameter inference

To infer the demographic parameters, PSMC uses an expectation-maximisation (EM) algorithm. Defining $f_\beta(i) = P(x_{1:i}, z_i = T_\beta)$ and $b_\alpha(i) = P(x_{i+1:L}|z_i = T_\alpha)$ as the forward and backward quantities respectively, we can calculate the expected transition matrix

$$A_{\beta\alpha} = \sum_i f_\beta(i) Q_{\beta\alpha}(\lambda_A) e_\alpha(x_{i+1}) b_\alpha(i+1) \tag{12}$$

where $Q^{\beta\alpha}(\lambda_A)$ denotes $Q(\alpha|\beta, \lambda_A)$, and update the demographic parameters from iteration v by maximising

$$F(\lambda_A^v) = \sum_{\alpha,\beta} log\left(Q_{\beta\alpha}(\lambda_A^v)\right) A_{\beta\alpha} \tag{13}$$

and setting

$$\lambda_A^{v+1} = argmax_{\lambda_A} F(\lambda_A^v) \tag{14}$$

2.3 *cobraa*'s HMM

The hidden states and emissions of *cobraa* are exactly the same as in PSMC. The emissions of *cobraa* are therefore given in equation (9). The transition probabilities however are different, because *cobraa* models changes in local ancestry according to a structured model, where as PSMC models according to an unstructured model. In the next section we thus derive the transition probabilities as a function of the structured model, by allowing migration in the SMC framework.

2.3.1 Transition probabilities under the structured model

In this section, we use the same notation for the SMC model as in 2.2.1: s is the given coalescence time for locus i, with next lowest time interval boundary τ_β; t is the new coalescence time for locus $i+1$ which is only dependent on locus i, with next lowest time interval boundary τ_α; u is the time of recombination which is strictly less than the minimum of (s,t) and greater than zero.

Under the SMC model, lineages can only either recombine or coalesce, resulting in a singular constraint that coalescence time must be greater than recombination time. If we introduce population structure, then lineages are also able to migrate. This induces some mutual exclusiveness, because certain types of coalescence event are disallowed. For example, in the *pulse* structure model, if we have a recombination at time $u < T_1$ where both solid lineages stay in population A but the floating lineage migrates to population B, then we cannot have any coalescence event at $t \in (T_1, T_2]$ because there are no solid lineages for the floating lineage to coalesce with. We therefore partition the structured transition matrix into 10 distinct Cases:

1. $t < T_1$ and $s > t$ with $s \in (t, \infty)$.
2. $T_1 \leq t < T_2$ and $T_1 \leq s < T_2$ with $s > t$.
3. $T_1 \leq t < T_2$ and $T_2 \leq s$.
4. $T_2 \leq t$ and $T_2 \leq s$ with $s > t$.
5. $t < T_1$ and $s < T_1$ with $t > s$.
6. $s < T_1$ and $T_1 \leq t < T_2$.
7. $T_1 \leq t < T_2$ and $T_1 \leq s < T_2$ with $t > s$.
8. $s < T_1$, $t > T_2$.
9. $T_1 \leq s < T_2$ and $t > T_2$.
10. $T_2 \leq t$ and $T_2 \leq s$ with $t > s$.

An illustration of these cases is shown in Supplementary Fig. 15. Note that Case 1 and Case 5 are the same as the panmictic case with $s > t$ and $t < s$, respectively. A possible configuration from Case 3 is shown in Supplementary Fig. 16, where at locus i the two solid lineages went through separate populations; a lineage recombines at time $u < T_1$ and migrates to population B, where it coalesces with the other lineage at time t. This results in the new genealogy at locus $i+1$.

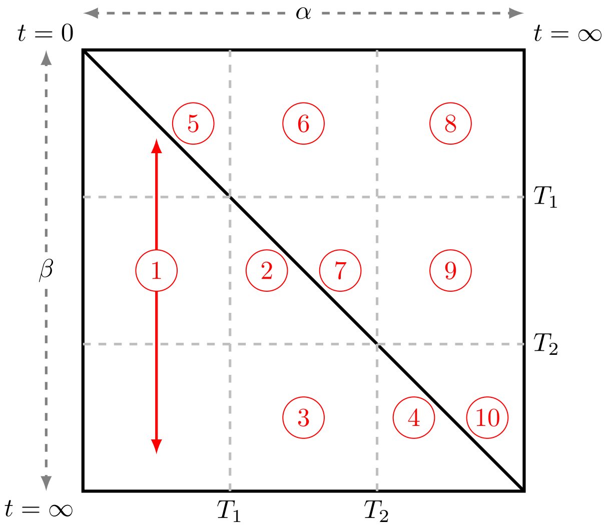

Supplementary Figure 15: Under the SMC model with a *pulse* model of population structure, given that a recombination has occurred, we partition the transition matrix in to 10 different possible coalescence events.

In what follows let $P_X(t|s) = P(z_{i+1} = t|z_i = s, \text{Case X})$ denote the probability of transitioning from TMRCA s to TMRCA t under the *pulse* migration model where s and t are bounded by the description given for Case X. After discretisation we write the transition matrix as $Q_X^{pulse}(\alpha|\beta)$ to denote $P_X(z_{i+1} = T_\alpha|z_i = T_\beta) = \int_{\tau_\alpha}^{\tau_{\alpha+1}} P_X(t|\langle t_\beta \rangle)dt$ where $\langle t_\beta \rangle$ is given by equation (10). The transition matrix under the *pulse* model Q^{pulse} is a function of λ_A, λ_B, γ, T_2, and T_1, which are, respectively, the discretised form of $1/N_A(t)$ and $1/N_B(t)$, admixture fraction, and the split and admixture time, though we frequently omit these for brevity.

We now walk through the derivation of Case 3. The other cases are given in the following sections. Two lineages combine at $s > T_2$ (the given current genealogy), so we must consider whether they both went through A, both through B, or one through A and one through B. Let $\mathbb{C}_3'^{AA}(s)$ denote two uncoalesced lineages at time s with both lineages having passed through population A, under the *pulse* migration model where s is bounded by the description for Case 3. Then

$$\begin{aligned} P(\mathbb{C}_3'^{AA}(s)) &= L_A(0, T_1)(1-\gamma)^2 L_A(T_1, T_2) L_A(T_2, s) \\ &= (1-\gamma)^2 L_A(0, s) \end{aligned} \tag{15}$$

Where the L_A terms are the probabilities of not coalescing (equation (6)) and the $(1-\gamma)^2$ term is the probability that both lineages stay in population A at time T_1. Similarly, we can write

$$P(\mathbb{C}_3'^{BB}(s)) = L_A(0, T_1)\gamma^2 L_B(T_1, T_2) L_A(T_2, s) \tag{16}$$

and

$$P(\mathbb{C}_3'^{AB}(s)) = 2\gamma(1-\gamma) L_A(0, T_1) L_A(T_2, s) \tag{17}$$

where $2\gamma(1-\gamma)$ is the probability that one lineage goes through A and the other through B at time T_1,

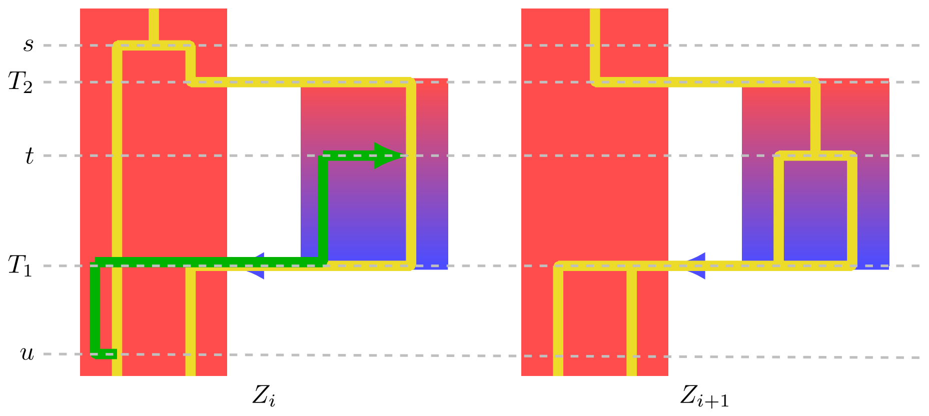

Supplementary Figure 16: Diagram of the SMC model under *pulse* population structure. Given the genealogy at locus i, a recombination occurs at time u and the floating lineage (green) migrates to population B at time T_1. The floating lineage then coalesces to the other solid lineage from which it recombined at time t, resulting in the new genealogy at locus $i+1$.

and the $L_A(T_1, T_2)$ or $L_B(T_1, T_2)$ term vanishes because two lineages can not coalesce if they are in different populations. Using ψ_3^{AA} to denote the probability of both lineages passing through population A, then by marginalising over $\mathbb{C}_3'^{AA}$, $\mathbb{C}_3'^{BB}$ and $\mathbb{C}_3'^{AB}$ we obtain

$$\begin{aligned}\psi_3^{AA} &= \frac{P(\mathbb{C}_3'^{AA})}{P(\mathbb{C}_3'^{AA}) + P(\mathbb{C}_3'^{BB}) + P(\mathbb{C}_3'^{AB})} \\ &= \frac{(1-\gamma)^2 L_A(T_1, T_2)}{(1-\gamma)^2 L_A(T_1, T_2) + \gamma^2 L_B(T_1, T_2) + 2\gamma(1-\gamma)}\end{aligned} \tag{18}$$

and similiarly for both lineages passing through B

$$\begin{aligned}\psi_3^{BB} &= \frac{P(\mathbb{C}_3'^{BB})}{P(\mathbb{C}_3'^{AA}) + P(\mathbb{C}_3'^{BB}) + P(\mathbb{C}_3'^{AB})} \\ &= \frac{\gamma^2 L_B(T_1, T_2)}{(1-\gamma)^2 L_A(T_1, T_2) + \gamma^2 L_B(T_1, T_2) + 2\gamma(1-\gamma)}\end{aligned} \tag{19}$$

and for one lineage through A and one through B

$$\begin{aligned}\psi_3^{AB} &= \frac{P(\mathbb{C}_3'^{AB})}{P(\mathbb{C}_3'^{AA}) + P(\mathbb{C}_3'^{BB}) + P(\mathbb{C}_3'^{AB})} \\ &= \frac{2\gamma(1-\gamma)}{(1-\gamma)^2 L_A(T_1, T_2) + \gamma^2 L_B(T_1, T_2) + 2\gamma(1-\gamma)}\end{aligned} \tag{20}$$

Now we consider the position of recombination, u, where strictly $0 \leq u \leq min(s, t)$. If $u \leq T_1$ then we must account for the probability of the floating lineage migrating to B or staying in A, and if there is one lineage in A and one in B then recombination can not occur after T_1 because we are conditioning on a

coalescent event in $(T_1, T_2]$ and $t \neq s$. Therefore, we have

$$P_3(t|s,R) = \frac{1}{s}\Bigg(\psi_3^{AA}\lambda_A(t)\Big(\int_0^{T_1} L_A(u,T_1)^2(1-\gamma)L_A(T_1,t)^2du + \int_{T_1}^{t} L_A(u,t)^2du\Big) + \psi_3^{BB}\lambda_B(t)\Big(\int_0^{T_1} L_A(u,T_1)^2\gamma L_B(T_1,t)^2du + \int_{T_1}^{t} L_B(u,t)^2du\Big) + \psi_3^{AB}\Big(\int_0^{T_1} L_A(u,T_1)^2\Big((1-\gamma)L_A(T_1,t)\lambda_A(t) + \gamma L_B(T_1,t)\lambda_B(t)\Big)\Big)\Bigg) \tag{21}$$

Recalling the piecewise constant assumption, we integrate out u by noticing that

$$\int_0^{T_1} L_A(u,T_1)^2du = \int_{\tau_0}^{\tau_1} L_A(u,\tau_1)^2L_A(\tau_1,T_1)^2du + \int_{\tau_1}^{\tau_2} L_A(u,\tau_2)^2L_A(\tau_2,T_1)^2du + \dots = \sum_{j=0}^{S-1}\frac{1}{2\lambda_{A_j}}\left(1-e^{-2\lambda_{A_j}\Delta_j}\right)L_A(\tau_{j+1},T_1)^2 \tag{22}$$

where $S \in \{1,\dots,D-1\}$ is the time index for T_1, $\Delta_j = \tau_{j+1}-\tau_j$ is the difference between neighbouring time interval boundaries, and the subscript j in λ_{A_j} denotes the j'th index of the vector λ_A, so $\lambda_{A_j} = 1/N_A(t)$ for $\tau_j \leq t < \tau_{j+1}$. We generalise this expression and define

$$K_A^1(\tau_a,\tau_b) = \int_{\tau_a}^{\tau_b} L_A(u,\tau_b)du = \sum_{j=a}^{b-1}\frac{1}{\lambda_{A_j}}\left(1-e^{\lambda_{A_j}\Delta_j}\right)L_A(\tau_{j+1},\tau_b) \tag{23}$$

$$K_A^2(\tau_a,\tau_b) = \int_{\tau_a}^{\tau_b} L_A(u,\tau_b)^2du = \sum_{j=a}^{b-1}\frac{1}{2\lambda_{A_j}}\left(1-e^{-2\lambda_{A_j}\Delta_j}\right)L_A(\tau_{j+1},\tau_b)^2 \tag{24}$$

so for example

$$\int_{T_1}^{t} L_A(u,t)^2du = \int_{T_1}^{t} L_A(u,\tau_\alpha)^2L_A(\tau_\alpha,t)^2du = \int_{T_1}^{\tau_\alpha} L_A(u,\tau_\alpha)^2L_A(\tau_\alpha,t)^2du + \int_{\tau_\alpha}^{t} L_A(u,t)^2du = K_A^2(T_1,\tau_\alpha)L_A(\tau_\alpha,t)^2 + \frac{1}{2\lambda_{A_\alpha}}\left(1-e^{-2\lambda_{A_\alpha}(t-\tau_\alpha)}\right) \tag{25}$$

Then, we can write

$$P_3(t|s,R) = \frac{1}{s}\Bigg(\psi_3^{AA}\lambda_A(t)\Bigg(K_A^2(\tau_0,T_1)(1-\gamma)L_A(T_1,t)^2 + K_A^2(T_1,\tau_\alpha)L_A(\tau_\alpha,t)^2 + \frac{1}{2\lambda_{A_\alpha}}\left(1-e^{-2\lambda_{A_\alpha}(t-\tau_\alpha)}\right)\Bigg)$$
$$+\psi_3^{BB}\lambda_B(t)\Bigg(K_A^2(\tau_0,T_1)\gamma L_B(T_1,t)^2 + K_B^2(T_1,\tau_\alpha)L_B(\tau_\alpha,t)^2 + \frac{1}{2\lambda_{B_\alpha}}\left(1-e^{-2\lambda_{B_\alpha}(t-\tau_\alpha)}\right)\Bigg)$$
$$+\psi_3^{AB}\frac{1}{2}\Bigg(K_A^2(\tau_0,T_1)\Big((1-\gamma)L_A(T_1,t)\lambda_A(t) + \gamma L_B(T_1,t)\lambda_B(t)\Big)\Bigg)\Bigg) \tag{26}$$

To get the transition probabilities, we must now integrate out s and t from $P_3(t|s,R)$, and take the discretised inverse population size parameters λ_A and λ_B. Similarly to Malaspinas et al. in [12] and Schiffels and Durbin in [13], we use the expected coalescence time $\langle t_\beta \rangle$ in interval β to discretise s (see equation (10)).

Define

$$J_A^1(\tau_\alpha) = \int_{\tau_\alpha}^{\tau_{\alpha+1}} L_A(\tau_\alpha,t)dt = \left(\frac{1}{\lambda_{A_\alpha}}\left(1-e^{-\lambda_{A_\alpha}\Delta_\alpha}\right)\right) \tag{27}$$

$$J_A^2(\tau_\alpha) = \int_{\tau_\alpha}^{\tau_{\alpha+1}} L_A(\tau_\alpha,t)^2dt = \left(\frac{1}{2\lambda_{A_\alpha}}\left(1-e^{-2\lambda_{A_\alpha}\Delta_\alpha}\right)\right) \tag{28}$$

and

$$H_A^1(\tau_\alpha) = \int_{\tau_\alpha}^{\tau_{\alpha+1}} \frac{1}{\lambda_{A_\alpha}}\left(1-e^{-\lambda_{A_\alpha}(t-\tau_\alpha)}\right)dt = \frac{1}{\lambda_{A_\alpha}}\left(\Delta_\alpha - \frac{1}{\lambda_{A_\alpha}}\left(1-e^{-\lambda_{A_\alpha}\Delta_\alpha}\right)\right) \tag{29}$$

$$H_A^2(\tau_\alpha) = \int_{\tau_\alpha}^{\tau_{\alpha+1}} \frac{1}{2\lambda_{A_\alpha}}\left(1-e^{-2\lambda_{A_\alpha}(t-\tau_\alpha)}\right)dt = \frac{1}{2\lambda_{A_\alpha}}\left(\Delta_\alpha - \frac{1}{2\lambda_{A_\alpha}}\left(1-e^{-2\lambda_{A_\alpha}\Delta_\alpha}\right)\right) \tag{30}$$

Then we have

$$Q_3^{pulse}(\alpha|\beta) = \int_{\tau_\alpha}^{\tau_{\alpha+1}} P(R|\langle t_\beta\rangle)P_3(t|\langle t_\beta\rangle,R)dt$$
$$= \frac{P(R|\langle t_\beta\rangle)}{\langle t_\beta\rangle}\Bigg(\psi_3^{AA}\lambda_{A_\alpha}\Big(K_A^2(\tau_0,T_1)(1-\gamma)L_A(T_1,\tau_\alpha)^2J_A^2(\tau_\alpha) + K_A^2(T_1,\tau_\alpha)J_A^2(\tau_\alpha) + H_A^2(\tau_\alpha)\Big) +$$
$$\psi_3^{BB}\lambda_{B_\alpha}\Big(K_A^2(\tau_0,T_1)\gamma L_B(T_1,\tau_\alpha)^2J_B^2(\tau_\alpha) + K_B^2(T_1,\tau_\alpha)J_B^2(\tau_\alpha) + H_B^2(\tau_\alpha)\Big) +$$
$$\psi_3^{AB}\frac{1}{2}\Big(K_A^2(\tau_0,T_1)\Big((1-\gamma)L_A(T_1,\tau_\alpha)J_A^1(\tau_\alpha)\lambda_{A_\alpha} + \gamma L_B(T_1,\tau_\alpha)J_B^1(\tau_\alpha)\lambda_{B_\alpha}\Big)\Big)\Bigg) \tag{31}$$

2.3.1.1 Case 1 $t < T_1$ and $s > t$ with $s \in (t, \infty)$

Case 1 is the same as panmixia with $s > t$, because $t < T_1$ so $u < T_1$ hence we do not need to consider the structured parameters. So

$$P_1(t|s) = \frac{P(R|s)}{s}\lambda_A(t)\left(\int_0^t L_A(u,t)^2 du\right) \tag{32}$$

then

$$\begin{aligned} Q_1^{pulse}(\alpha|\beta) &= \int_{\tau_\alpha}^{\tau_{\alpha+1}} P_1(t|\langle t_\beta\rangle)dt \\ &= \frac{P(R|\langle t_\beta\rangle)}{\langle t_\beta\rangle}\lambda_{A_\alpha}\left(K_A^2(T_0,\tau_\alpha)J_A^2(\tau_\alpha) + H_A^2(\tau_\alpha)\right) \end{aligned} \tag{33}$$

2.3.1.2 Case 2 $T_1 \le t < T_2$ and $T_1 \le s < T_2$ with $s > t$

Case 2 is very similar to Case 3, though because we are conditioning on $s, t \in (T_1, T_2]$ we cannot have the solid lineages in separate populations therefore ψ_2^{AB}=0. So

$$\begin{aligned} P_2(t|s) = \frac{P(R|s)}{s}\Bigg(&\psi_{2_\beta}^{AA}\lambda_A(t)\Big(\int_0^{T_1} L_A(u,T_1)^2(1-\gamma)L_A(T_1,t)^2 du + \int_{T_1}^t L_A(u,t)^2 du\Big) + \\ &\psi_{2_\beta}^{BB}\lambda_B(t)\Big(\int_0^{T_1} L_A(u,T_1)^2\gamma L_B(T_1,t)^2 du + \int_{T_1}^t L_B(u,t)^2 du\Big)\Bigg) \end{aligned} \tag{34}$$

where

$$\psi_{2_\beta}^{AA} = \frac{\lambda_{A_\beta}(1-\gamma)^2 L_A(T_1,\langle t_\beta\rangle)}{\lambda_{A_\beta}(1-\gamma)^2 L_A(T_1,\langle t_\beta\rangle)) + \lambda_{B_\beta}\gamma^2 L_B(T_1,\langle t_\beta\rangle))} \tag{35}$$

and

$$\psi_{2_\beta}^{BB} = \frac{\lambda_{B_\beta}\gamma^2 L_A(T_1,\langle t_\beta\rangle))}{\lambda_{A_\beta}(1-\gamma)^2 L_A(T_1,\langle t_\beta\rangle)) + \lambda_{B_\beta}\gamma^2 L_B(T_1,\langle t_\beta\rangle))} \tag{36}$$

then integrating out u, t, and s yields

$$\begin{aligned} Q_2^{pulse}(\alpha|\beta) &= \int_{\tau_\alpha}^{\tau_{\alpha+1}} P_2(t|\langle t_\beta\rangle)dt \\ &= \frac{P(R|\langle t_\beta\rangle}{\langle t_\beta\rangle}\Bigg(\psi_{2_\beta}^{AA}\lambda_{A_\alpha}\Big(K_A^2(T_0,T_1)(1-\gamma)L_A(T_1,\tau_\alpha)^2 J_A^2(\tau_\alpha) + K_A^2(T_1,\tau_\alpha)J_A^2(\tau_\alpha) + H_A^2(\tau_\alpha)\Big) + \\ &\qquad \psi_{2_\beta}^{BB}\lambda_{B_\alpha}\Big(K_A^2(T_0,T_1)(\gamma)L_B(T_1,\tau_\alpha)^2 J_B^2(\tau_\alpha) + K_B^2(T_1,\tau_\alpha)J_B^2(\tau_\alpha) + H_B^2(\tau_\alpha)\Big)\Bigg) \end{aligned} \tag{37}$$

2.3.1.3 Case 3 $\quad T_1 \le t < T_2$ and $T_2 \le s$

The derivation for Case 3 is given above, but for continuity we restate here

$$
\begin{aligned}
Q_3^{pulse}(\alpha|\beta) &= \int_{\tau_\alpha}^{\tau_{\alpha+1}} P(R|\langle t_\beta\rangle) P_3(t|\langle t_\beta\rangle, R)dt \\
&= \frac{P(R|\langle t_\beta\rangle)}{\langle t_\beta\rangle}\Bigg(\psi_3^{AA}\lambda_{A_\alpha}\Big(K_A^2(\tau_0,T_1)(1-\gamma)L_A(T_1,\tau_\alpha)^2 J_A^2(\tau_\alpha) + K_A^2(T_1,\tau_\alpha)J_A^2(\tau_\alpha) + H_A^2(\tau_\alpha)\Big)+ \\
&\qquad \psi_3^{BB}\lambda_{B_\alpha}\Big(K_A^2(\tau_0,T_1)\gamma L_B(T_1,\tau_\alpha)^2 J_B^2(\tau_\alpha) + K_B^2(T_1,\tau_\alpha)J_B^2(\tau_\alpha) + H_B^2(\tau_\alpha)\Big)+ \\
&\qquad \psi_3^{AB}\frac{1}{2}\Big(K_A^2(\tau_0,T_1)\Big((1-\gamma)L_A(T_1,\tau_\alpha)J_A^1(\tau_\alpha)\lambda_{A_\alpha} + \gamma L_B(T_1,\tau_\alpha)J_B^1(\tau_\alpha)\lambda_{B_\alpha}\Big)\Big)\Bigg)
\end{aligned} \tag{38}
$$

2.3.1.4 Case 4 $\quad T_2 \le t$ and $T_2 \le s$ with $s > t$

We have

$$
\begin{aligned}
P_4(t|s,R) = \frac{1}{s}\lambda_A(t)\Bigg(&\psi_4^{AA}\Big(\int_0^{T_1} L_A(u,T_1)^2 du\Big(\gamma + (1-\gamma)L_A(T_1,T_2)^2\Big)L_A(T_2,t)^2 + \\
&\qquad \int_{T_1}^{T_2} L_A(u,T_2)^2 du Ł_A(T_2,t)^2 + \int_{T_2}^{t} L_A(u,t)^2 du\Big)+ \\
&\psi_4^{BB}\Big(\int_0^{T_1} L_A(u,T_1)^2 du\Big((1-\gamma) + \gamma L_B(T_1,T_2)^2\Big)L_A(T_2,t)^2 + \\
&\qquad \int_{T_1}^{T_2} L_B(u,T_2)^2 du Ł_A(T_2,t)^2 + \int_{T_2}^{t} L_A(u,t)^2 du\Big)+ \\
&\psi_4^{AB}\Big(\int_0^{T_1} L_A(u,T_1)^2 du\Big((1-\gamma)L_A(T_1,T_2) + \gamma L_B(T_1,T_2)\Big)L_A(T_2,t)^2 + \\
&\qquad \frac{1}{2}\int_{T_1}^{T_2}\Big(L_A(u,T_2) + L_B(u,T_2)\Big)du Ł_A(T_2,t)^2 + \int_{T_2}^{t} L_A(u,t)^2 du\Big)\Bigg)
\end{aligned} \tag{39}
$$

where

$$
\psi_4^{AA} = \frac{(1-\gamma)^2 L_A(T_1,T_2)}{(1-\gamma)^2 L_A(T_1,T_2) + \gamma^2 L_B(T_1,T_2) + 2\gamma(1-\gamma)}
$$

$$
\psi_4^{BB} = \frac{\gamma^2 L_B(T_1,T_2)}{(1-\gamma)^2 L_A(T_1,T_2) + \gamma^2 L_B(T_1,T_2) + 2\gamma(1-\gamma)}
$$

$$
\psi_4^{AB} = \frac{2\gamma(1-\gamma)}{(1-\gamma)^2 L_A(T_1,T_2) + \gamma^2 L_B(T_1,T_2) + 2\gamma(1-\gamma)} \tag{40}
$$

From which we obtain

$$Q_4^{pulse}(\alpha|\beta) = \int_{\tau_\alpha}^{\tau_{\alpha+1}} P_4(R|\langle t_\beta\rangle)P_4(t|\langle t_\beta\rangle)dt$$

$$= \frac{P(R|\langle t_\beta\rangle)}{\langle t_\beta\rangle}\lambda_{A_\alpha}\Bigg(\psi_4^{AA}\Bigg(K_A^2(T_0,T_1)\big(\gamma+(1-\gamma)L_A(T_1,T_2)^2\big)L_A(T_2,\tau_\alpha)^2J_A^2(\tau_\alpha)+$$
$$K_A^2(T_1,T_2)L_A(T_2,\tau_\alpha)^2J_A^2(\tau_\alpha)+K_A^2(T_2,\tau_\alpha)J_A^2(\tau_\alpha)+H_A^2(\tau_\alpha)\Bigg)$$
$$+\psi_4^{BB}\Bigg(K_A^2(T_0,T_1)\big((1-\gamma)+\gamma L_B(T_1,T_2)^2\big)L_A(T_2,\tau_\alpha)^2J_A^2(\tau_\alpha)+$$
$$K_B^2(T_1,T_2)L_A(T_2,\tau_\alpha)^2J_A^2(\tau_\alpha)+K_A^2(T_2,\tau_\alpha)J_A^2(\tau_\alpha)+H_A^2(\tau_\alpha)\Bigg)$$
$$+\psi_4^{AB}\Bigg(K_A^2(T_0,T_1)\big((1-\gamma)L_A(T_1,T_2)+\gamma L_B(T_1,T_2)\big)L_A(T_2,\tau_\alpha)^2J_A^2(\tau_\alpha)+$$
$$\frac{1}{2}\big(K_A^1(T_1,T_2)+K_B^1(T_1,T_2)\big)L_A(T_2,\tau_\alpha)^2J_A^2(\tau_\alpha)+K_A^2(T_2,\tau_\alpha)J_A^2(\tau_\alpha)+H_\alpha^2(\tau_\alpha)\Bigg)\Bigg) \tag{41}$$

2.3.1.5 Case 5 $\quad t < T_1$ and $s < T_1$ with $t > s$

Case 5 is the same as panmixia with $t > s$, because $t < T_1$ so $u < T_1$ hence we do not need to consider the structured parameters. So

$$P_5(t|s,R) = \frac{1}{s}\lambda_A(t)\left(\int_0^s L_A(u,s)^2du L_A(s,t)\right) \tag{42}$$

then

$$Q_5^{pulse}(\alpha|\beta) = \int_{\tau_\alpha}^{\tau_{\alpha+1}} P(R|\langle t_\beta\rangle)P_5(t|\langle t_\beta\rangle)dt$$
$$= \frac{P(R|\langle t_\beta\rangle}{\langle t_\beta\rangle}\lambda_{A_\alpha}L_A(s,\tau_\alpha)J_A^1(\tau_\alpha)\Bigg(K_A^2(\tau_0,\tau_\beta)L_A(\tau_\beta,\langle t_\beta\rangle)^2+J_A^2(\tau_\beta,\langle t_\beta\rangle)\Bigg) \tag{43}$$

2.3.1.6 Case 6 $\quad s < T_1$ and $T_1 \leq t < T_2$

For Case 6, two lineages have coalesced at $s < T_1$ so we only consider the possibility of one lineage migrating at time T_1, then $\psi_6^A = (1-\gamma)$ and $\psi_6^B = \gamma$. So

$$P_6(t|s,R) = \frac{1}{s}\Bigg(\psi_6^A\lambda_A(t)\Big(\int_0^s L_A(u,s)^2du L_A(s,T_1)(1-\gamma)L_A(T_1,t)\Big)+$$
$$\psi_6^B\lambda_A(t)\Big(\int_0^s L_A(u,s)^2du L_A(s,T_1)\gamma L_B(T_1,t)\Big)\Bigg) \tag{44}$$

then

$$Q_6^{pulse}(\alpha|\beta) = \int_{\tau_\alpha}^{\tau_{\alpha+1}} P(R|\langle t_\beta\rangle)P_6(t|\langle t_\beta\rangle, R)dt$$

$$= \frac{P(R|\langle t_\beta\rangle)}{\langle t_\beta\rangle}\Bigg(\psi_6^A\lambda_{A_\alpha}\Big(L_A(\langle t_\beta\rangle, T_1)(1-\gamma)L_A(T_1,\tau_\alpha)J_A^1(\tau_\alpha)\left(K_A^2(T_0,\tau_\beta)L_A(\tau_\beta,\langle t_\beta\rangle)^2 + J_A^2(\tau_\beta,\langle t_\beta\rangle)\right)\Big)+$$

$$\psi_6^B\lambda_{B_\alpha}\Big(L_A(\langle t_\beta\rangle, T_1)\gamma L_B(T_1,\tau_\alpha)J_B^1(\tau_\alpha)\big(K_A^2(T_0,\tau_\beta)L_A(\tau_\beta,\langle t_\beta\rangle)^2 + J_A^2(\tau_\beta,\langle t_\beta\rangle)\big)\Big)\Bigg) \qquad (45)$$

2.3.1.7 Case 7 $T_1 \leq t < T_2$ and $T_1 \leq s < T_2$ with $t > s$

For Case 7 we have

$$P_7(t|s,R) = \frac{1}{s}\Bigg(\psi_{7_\beta}^{AA}\lambda_A(t)\Big(\int_0^{T_1} L_A(u,T_1)^2du(1-\gamma)L_A(T_1,s)^2L_A(s,t) + \int_{T_1}^{s} L_A(u,s)^2L_A(s,t)du\Big)+$$

$$\psi_{7_\beta}^{BB}\lambda_B(t)\Big(\int_0^{T_1} L_A(u,T_1)^2du\gamma L_B(T_1,s)^2L_B(s,t) + \int_{T_1}^{s} L_B(u,s)^2L_B(s,t)du\Big)\Bigg) \qquad (46)$$

where

$$\psi_{7_\beta}^{AA} = \frac{\lambda_{A_\beta}(1-\gamma)^2L_A(T_1,\langle t_\beta\rangle)}{\lambda_{A_\beta}(1-\gamma)^2L_A(T_1,\langle t_\beta\rangle) + \lambda_{B_\beta}\gamma^2L_B(T_1,\langle t_\beta\rangle)} \qquad (47)$$

$$\psi_{7_\beta}^{BB} = \frac{\lambda_{B_\beta}\gamma^2L_B(T_1,\langle t_\beta\rangle)}{\lambda_{A_\beta}(1-\gamma)^2L_A(T_1,\langle t_\beta\rangle) + \lambda_{B_\beta}\gamma^2L_B(T_1,\langle t_\beta\rangle)} \qquad (48)$$

So

$$Q_7^{pulse}(\alpha|\beta) = \int_{\tau_\alpha}^{\tau_{\alpha+1}} P(R|\langle t_\beta\rangle)P_7(t|\langle t_\beta\rangle, R)dt$$

$$= \frac{P_7(R|\langle t_\beta\rangle)}{\langle t_\beta\rangle}\Bigg(\psi_{7_\beta}^{AA}\lambda_{A_\alpha}\Bigg(L_A(\langle t_\beta\rangle,\tau_\alpha)J_A^1(\tau_\alpha)\Bigg(K_A^2(T_0,T_1)(1-\gamma)L_A(T_1,\langle t_\beta\rangle)^2+$$

$$K_A^2(T_1,\tau_\beta)L_A(\tau_\beta,\langle t_\beta\rangle)^2 + J_A^2(\tau_\beta,\langle t_\beta\rangle)\Bigg)\Bigg)+$$

$$\psi_{7_\beta}^{BB}\lambda_{B_\alpha}\Bigg(L_B(\langle t_\beta\rangle,\tau_\alpha)J_B^1(\tau_\alpha)\Bigg(K_A^2(T_0,T_1)\gamma L_B(T_1,\langle t_\beta\rangle)^2+$$

$$K_B^2(T_1,\tau_\beta)L_B(\tau_\beta,\langle t_\beta\rangle)^2 + J_B^2(\tau_\beta,\langle t_\beta\rangle)\Bigg)\Bigg)\Bigg) \qquad (49)$$

2.3.1.8 Case 8 $s < T_1$ and $t > T_2$

For Case 8 we have

$$P_8(t|s,R) = \frac{1}{s}\lambda_A(t)\Bigg(\psi_8^A\left(\int_0^s L_A(u,s)^2 du L_A(s,T_1)L_A(T_2,t)\left((1-\gamma)L_A(T_1,T_2)+\gamma\right)\right)+ \psi_8^B\left(\int_0^s L_A(u,s)^2 du L_A(s,T_1)L_A(T_2,t)\left(\gamma L_B(T_1,T_2)+(1-\gamma)\right)\right)\Bigg) \tag{50}$$

where $\psi_8^A = (1-\gamma)$ and $\psi_8^B = \gamma$. Then

$$\begin{aligned} Q_8^{pulse}(\alpha|\beta) &= \int_{\tau_\alpha}^{\tau_{\alpha+1}} P(R|\langle t_\beta\rangle)P_8(t|\langle t_\beta\rangle,R)dt \\ &= \frac{P(R|\langle t_\beta\rangle)}{\langle t_\beta\rangle}\lambda_{A_\alpha}\Bigg(\psi_8^A\bigg(\Big(K_A^2(T_0,\tau_\beta)L_A(\tau_\beta,\langle t_\beta\rangle)^2 + J_A^2(\tau_\beta,\langle t_\beta\rangle)\Big)L_A(\langle t_\beta\rangle,T_1)\times \\ &\qquad \Big((1-\gamma)L_A(T_1,T_2)+\gamma\Big)L_A(T_2,\tau_\alpha)J_A^1(\tau_\alpha)\bigg)+ \\ &\qquad \psi_8^B\bigg(\Big(K_A^2(T_0,\tau_\beta)L_A(\tau_\beta,\langle t_\beta\rangle)^2 + J_A^2(\tau_\beta,\langle t_\beta\rangle)\Big)L_A(\langle t_\beta\rangle,T_1)\times \\ &\qquad \Big(\gamma L_B(T_1,T_2)+(1-\gamma)\Big)L_A(T_2,\tau_\alpha)J_A^1(\tau_\alpha)\bigg)\Bigg) \end{aligned} \tag{51}$$

2.3.1.9 Case 9 $T_1 \le s < T_2$ and $t > T_2$

For Case 9 we have

$$\begin{aligned} P_9(t|s,R) = \frac{1}{s}\lambda_A(t)\Bigg(&\psi_{9_\beta}^{AA}\bigg(L_A(T_2,t)\Big(\int_0^{T_1} L_A(u,T_1)^2 du\Big((1-\gamma)L_A(T_1,s)^2 L_A(s,T_2)+\gamma\Big)+ \\ &\int_{T_1}^s L_A(u,s)^2 du L_A(s,T_2)\Big)\bigg)+ \\ &\psi_{9_\beta}^{BB}\bigg(L_A(T_2,t)\Big(\int_0^{T_1} L_A(u,T_1)^2 du\Big(\gamma L_B(T_1,s)^2 L_B(s,T_2)+(1-\gamma)\Big)+ \\ &\int_{T_1}^s L_B(u,s)^2 du L_B(s,T_2)\Big)\bigg)\Bigg) \end{aligned} \tag{52}$$

where

$$\psi_{9_\beta}^{AA} = \frac{\lambda_A(s)(1-\gamma)^2 L_A(T_1,s)}{\lambda_A(s)(1-\gamma)^2 L_A(T_1,s)+\lambda_B(s)\gamma^2 L_B(T_1,s)} \tag{53}$$

and

$$\psi_{9_\beta}^{BB} = \frac{\lambda_B(s)\gamma^2 L_B(T_1,s)}{\lambda_A(s)(1-\gamma)^2 L_A(T_1,s)+\lambda_B(s)\gamma^2 L_B(T_1,s)} \tag{54}$$

So

$$Q_9^{pulse}(\alpha|\beta) = \int_{\tau_\alpha}^{\tau_{\alpha+1}} P(R|\langle t_\beta\rangle)P_9(t|\langle t_\beta\rangle, R)dt$$

$$= \frac{P(R|\langle t_\beta\rangle)}{\langle t_\beta\rangle}\lambda_{A_\alpha}\Bigg(\psi_{9_\beta}^{AA}\Big(L_A(T_2,\tau_\alpha)J_A^1(\tau_\alpha)\Big(K_A^2(T_0,T_1)\big((1-\gamma)L_A(T_1,\langle t_\beta\rangle)^2L_A(\langle t_\beta\rangle,T_2)+\gamma\big)+ \big(K_A^2(T_1,\tau_\beta)L_A(\tau_\beta,\langle t_\beta\rangle)^2+J_A^2(\tau_\beta,\langle t_\beta\rangle)\big)L_A(\langle t_\beta\rangle,T_2)\Big)+ \psi_{9_\beta}^{BB}\Big(L_A(T_2,\tau_\alpha)J_A^1(\tau_\alpha)\Big(K_A^2(T_0,T_1)\big(\gamma L_B(T_1,\langle t_\beta\rangle)^2L_B(\langle t_\beta\rangle,T_2)+(1-\gamma)\big)+ \big(K_B^2(T_1,\tau_\beta)L_B(\tau_\beta,\langle t_\beta\rangle)^2+J_B^2(\tau_\beta,\langle t_\beta\rangle)\big)L_B(\langle t_\beta\rangle,T_2)\Big)\Bigg) \tag{55}$$

2.3.1.10 Case 10 $\quad T_2 \leq t$ and $T_2 \leq s$ with $t > s$

For Case 10 we have

$$P_{10}(t|s,R) = \frac{1}{s}\lambda_A(t)\Bigg(\psi_{10}^{AA}\Big(\int_0^{T_1} L_A(u,T_1)^2du L_A(T_2,s)^2L_A(s,t)\Big((1-\gamma)L_A(T_1,T_2)^2+\gamma\Big)+ \int_{T_1}^{T_2} L_A(u,T_2)^2du L_A(T_2,s)^2L_A(s,t)+\int_{T_2}^{s} L_A(u,s)^2du L_A(s,t)\Big)+ \psi_{10}^{BB}\Big(\int_0^{T_1} L_A(u,T_1)^2du L_A(T_2,s)^2L_A(s,t)\Big(\gamma L_B(T_1,T_2)^2+(1-\gamma)\Big)+ \int_{T_1}^{T_2} L_B(u,T_2)^2du L_A(T_2,s)^2L_A(s,t)+\int_{T_2}^{s} L_A(u,s)^2du L_A(s,t)\Big)+ \psi_{10}^{AB}\Big(\int_0^{T_1} L_A(u,T_1)^2du\Big(\gamma L_B(T_1,T_2)+(1-\gamma)L_A(T_1,T_2)\Big)L_A(T_2,s)^2L_A(s,t)+ \frac{1}{2}\Big(\int_{T_1}^{T_2} L_B(u,T_2)du+\int_{T_1}^{T_2} L_A(u,T_2)du\Big)L_A(T_2,s)^2L_A(s,t)+ \int_{T_2}^{s} L_A(u,s)^2du L_A(s,t)\Big)\Bigg) \tag{56}$$

where

$$\psi_{10}^{AA} = \frac{(1-\gamma)^2L_A(T_1,T_2)}{(1-\gamma)^2L_A(T_1,T_2)+\gamma^2L_B(T_1,T_2)+2\gamma(1-\gamma)} \tag{57}$$

$$\psi_{10}^{BB} = \frac{\gamma^2L_B(T_1,T_2)}{(1-\gamma)^2L_A(T_1,T_2)+\gamma^2L_B(T_1,T_2)+2\gamma(1-\gamma)} \tag{58}$$

$$\psi_{10}^{AB} = \frac{2\gamma(1-\gamma)}{(1-\gamma)^2L_A(T_1,T_2)+\gamma^2L_B(T_1,T_2)+2\gamma(1-\gamma)} \tag{59}$$

so

$$
\begin{aligned}
Q_{10}^{pulse}(\alpha|\beta) &= \int_{\tau_\alpha}^{\tau_{\alpha+1}} P(R|\langle t_\beta\rangle)P_{10}(t|\langle t_\beta\rangle, R)dt \\
= \frac{P(R|\langle t_\beta\rangle)}{\langle t_\beta\rangle}\lambda_{A_\alpha}\Bigg(&\psi_{10}^{AA}\Bigg(K_A^2(T_0,T_1)L_A(T_2,\langle t_\beta\rangle)^2 L_A(\langle t_\beta\rangle,\tau_\alpha)J_A^1(\tau_\alpha)\Big((1-\gamma)L_A(T_1,T_2)^2+\gamma\Big)+ \\
&K_A^2(T_1,T_2)L_A(T_2,\langle t_\beta\rangle)^2 L_A(\langle t_\beta\rangle,\tau_\alpha)J_A^1(\tau_\alpha)+ \\
&\Big(K_A^2(T_2,\tau_\beta)L_A(\tau_\beta,\langle t_\beta\rangle)^2 + J_A^2(\tau_\beta,\langle t_\beta\rangle)\Big)L_A(\langle t_\beta\rangle,\tau_\alpha)J_A^1(\tau_\alpha)\Bigg)+ \\
&\psi_{10}^{BB}\Bigg(K_A^2(T_0,T_1)L_A(T_2,\langle t_\beta\rangle)^2 L_A(\langle t_\beta\rangle,\tau_\alpha)J_A^1(\tau_\alpha)\Big(\gamma L_B(T_1,T_2)^2+(1-\gamma)\Big)+ \\
&K_B^2(T_1,T_2)L_A(T_2,\langle t_\beta\rangle)^2 L_A(\langle t_\beta\rangle,\tau_\alpha)J_A^1(\tau_\alpha)+ \\
&\Big(K_A^2(T_2,\tau_\beta)L_A(\tau_\beta,\langle t_\beta\rangle)^2 + J_A^2(\tau_\beta,\langle t_\beta\rangle)\Big)L_A(\langle t_\beta\rangle,\tau_\alpha)J_A^1(\tau_\alpha)\Bigg)+ \\
&\psi_{10}^{AB}\Bigg(K_A^2(T_0,T_1)L_A(T_2,\langle t_\beta\rangle)^2 L_A(\langle t_\beta\rangle,\tau_\alpha)J_A^1(\tau_\alpha)\Big((1-\gamma)L_A(T_1,T_2)+\gamma L_B(T_1,T_2)\Big)+ \\
&\frac{1}{2}\Big(K_A^1(T_1,T_2)+K_B^1(T_1,T_2)\Big)L_A(T_2,\langle t_\beta\rangle)^2 L_A(\langle t_\beta\rangle,\tau_\alpha)J_A^1(\tau_\alpha)+ \\
&\Big(K_A^2(T_2,\tau_\beta)L_A(\tau_\beta,\langle t_\beta\rangle)^2 + J_A^2(\tau_\beta,\langle t_\beta\rangle)\Big)L_A(\langle t_\beta\rangle,\tau_\alpha)J_A^1(\tau_\alpha)\Bigg)\Bigg)
\end{aligned} \tag{60}
$$

2.3.1.11 Transitions to self

Combining all the different Cases, we can write

$$Q^{pulse}(\alpha|\beta) = \sum_{i=1}^{10} Q_i^{pulse}(\alpha|\beta) \tag{61}$$

then use the law of total probability to describe the probability that a hidden state transitions to itself (i.e. the probability that no recombination occurred, or after a recombination the lineage coalesced with itself):

$$Q^{pulse}(\alpha|\alpha) = 1 - \sum_{i\in(1\ldots,D)}^{D} Q^{pulse}(i|\alpha)\mathbb{1}_{i\neq\alpha} \tag{62}$$

2.3.2 Demonstration of correctness

To validate the transition probabilities for a *pulse* structured model under the SMC model, we compare our theory against simulations using *msprime* [14]. In this section, we denote empirical summary statistics from the simulated data with a circumflex and we omit the *pulse* superscript and the function's demographic parameters.

With a specific demographic model (defined by parameters $\lambda_A, \lambda_B, \gamma, T_1, T_2, D, \omega, T_{max}$), and simulating with *msprime* we can:

1. Record the sequence of marginal coalescence events Z from the ARG. Denote its length by N

2. Discretise the marginal coalescence times into the HMM's corresponding time states T, which is defined by D, ω, T_{max}: $\bar{Z} = \lfloor Z \rfloor_T$

3. Treating $\bar{Z}$ as a Markov chain, we can calculate a matrix which counts the number of transitions from state β to α

$$\hat{A}_{\beta\alpha} = \sum_{i=1}^{N} \mathbb{1}_{\{\bar{Z}_i=\beta, \bar{Z}_{i+1}=\alpha\}} \tag{63}$$

where $\mathbb{1}_E$ is 1 if event E is true and 0 otherwise.

4. From the matrix of counts we can calculate the MLE of the transition matrix for sequence $\bar{Z}$

$$\hat{Q}_{\beta\alpha} = \frac{\hat{A}_{\beta\alpha}}{\sum_{i=1}^{D} \hat{A}_{\beta i}}. \tag{64}$$

From $\hat{A}$ we can calculate the proportion of time spent in each time state with

$$\hat{\pi}_{\alpha} = \frac{\sum_{i=1}^{D} \hat{A}_i}{\sum_{i,j \in (1 \ldots D)} \hat{A}_{ij}} \tag{65}$$

. We can also compare $\hat{Q}$ with the probabilities given by the theoretical matrix Q.

With an arbitrary demographic model

Model 2.1 *pulse structured model*

$$D = 32, \omega = 0.1, T_{max} = 40, 2\bar{N}_A = 1e+04, \gamma = 0.3, T_1 = 10, T_2 = 18$$
$$\lambda_A = \mathbf{1}$$
$$\lambda_B = \begin{cases} 1 & i \notin \{T_1, \ldots, T_2\} \\ 2 & i \in \{T_1, \ldots, T_2\} \end{cases}$$

We simulate ∼2e+7 transitions under the Hudson [15, 16] and SMC [5, 7] model with *msprime* [14] and compare this to *cobraa*'s theoretical transition matrix Q^{pulse} (we use Marjoram and Wall's SMC model for simulation, which *msprime* labels "SMCprime"). When discretising time, we can either take the expected coalescence time in each interval, $\langle t_\beta \rangle$, or the midpoint between boundaries. We calculate the difference between the median simulated coalescence time in each time window and either the expected coalescence time or midpoint in Supplementary Fig. 17a, observing that each gets increasingly inaccurate as the time windows get larger in ancient time.

In Supplementary Fig. 17b and 17c we plot the proportion of time spent in each state for a *pulse* structured model, simulated with Hudson $\hat{\pi}^{Hudson}$ and SMC $\hat{\pi}^{SMC}$, against the theoretical stationary distribution π as described in section 2.3.1. These indicate that the Hudson and SMC simulation model are almost indistinguishable from each other, demonstrating the accuracy of the SMC [8]. Moreover the theoretical predictions from *cobraa* are practically identical to the Hudson or SMC simulations except in the most ancient time states where there are larger residuals. These larger residuals are a consequence of the discretisation of time and not a limitation of the theoretical predictions, as the sign of the residuals in ancient

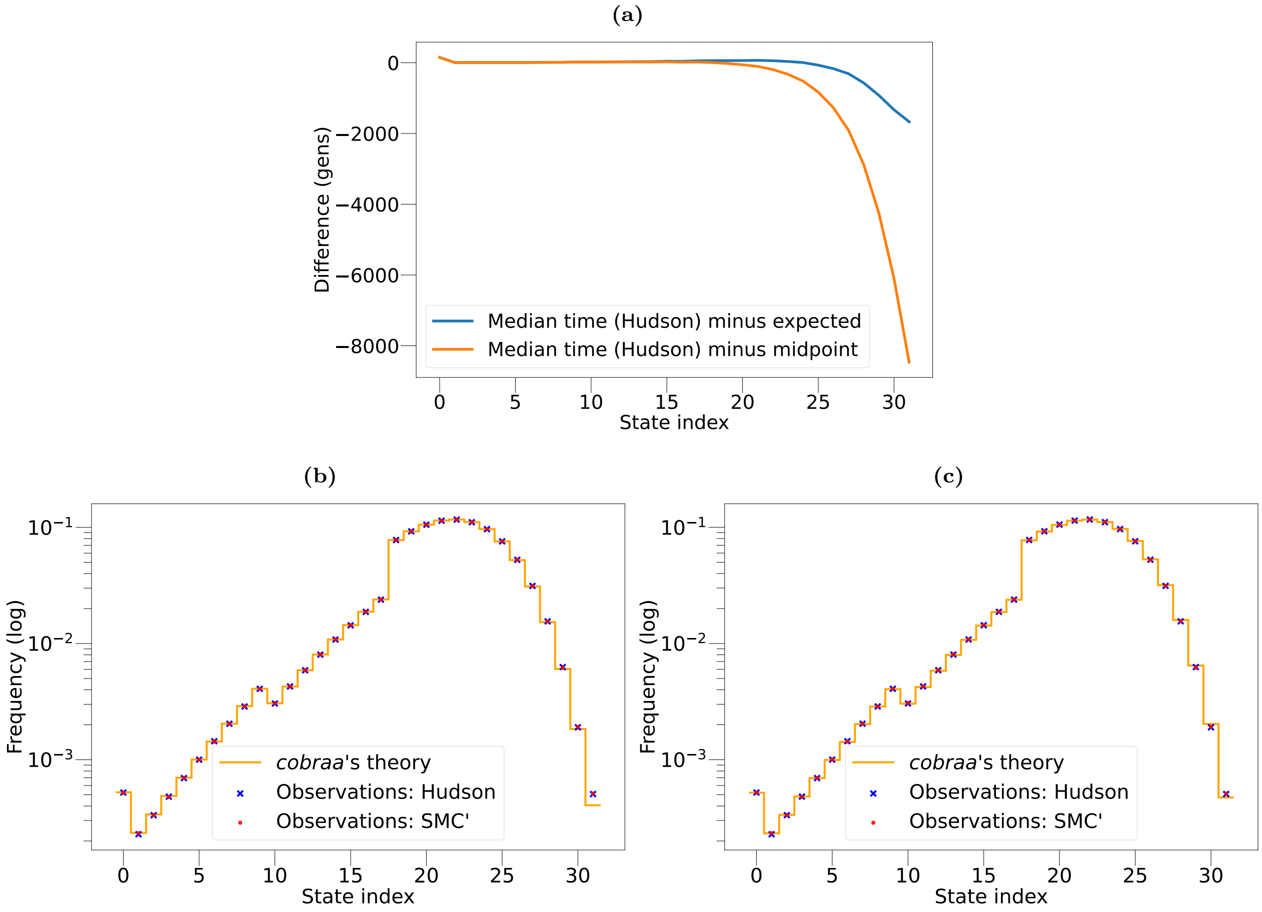

Supplementary Figure 17: **a)** Difference between the expected coalescence time $\langle t_\beta \rangle$ (blue) or the midpoint of each time interval $(\tau_\beta + \tau_{\beta+1})/2$ (orange) against the median of all simulated coalescence events that occurred in that interval, from Hudson's model. Each approximation becomes more inaccurate as the size of the time interval boundary gets larger. **b)** and **c)** The (log) proportion of time spent in each state, according to a simulation with the Hudson (blue star) and SMC (red point) model, with the theoretical proportion from *cobraa* (orange line) as described by $\pi(t)$. (b) Uses the expected coalescence time in each interval, where as (c) uses the midpoint of each interval. We use "SMC" to denote Marjoram and Wall's modified SMC model, which *msprime* labels as "SMCprime".

time states flips between 17b and 17c.

In Supplementary Fig. 18 we visualise the relative difference between *cobraa*'s theoretical transition matrix and the MLE of the transition matrix from a simulation under Hudson $\xi = (\hat{Q}^{Hudson} - Q^{pulse})/Q^{pulse}$, within $\pm 5\%$ variation. For ancient transitions we see larger residuals, though again these are a consequence of time discritisation, as seen from changing between using the expected coalescence time (Supplementary Fig. 18a) or the midpoint (Fig. 18b). The remaining residuals are consistent with sampling noise. Furthermore we can visualise a particular row of ξ, to see how $\hat{Q}^{Hudson}$, $\hat{Q}^{SMC}$ and Q differ. Setting $\beta = 15$ as a time state in the structured period, we visualise these distributions in Supplementary Fig. 18c. It appears that $\hat{Q}^{Hudson}_{15,i}$ and $\hat{Q}^{SMC}_{15,i}$ are virtually indistinguishable from each other, except from particularly recent or ancient time states. However, the number of transitions from these states are low and hence the MLE estimates may not be especially reliable - for example, at state 31 there are only $\sim$36 transitions recorded.

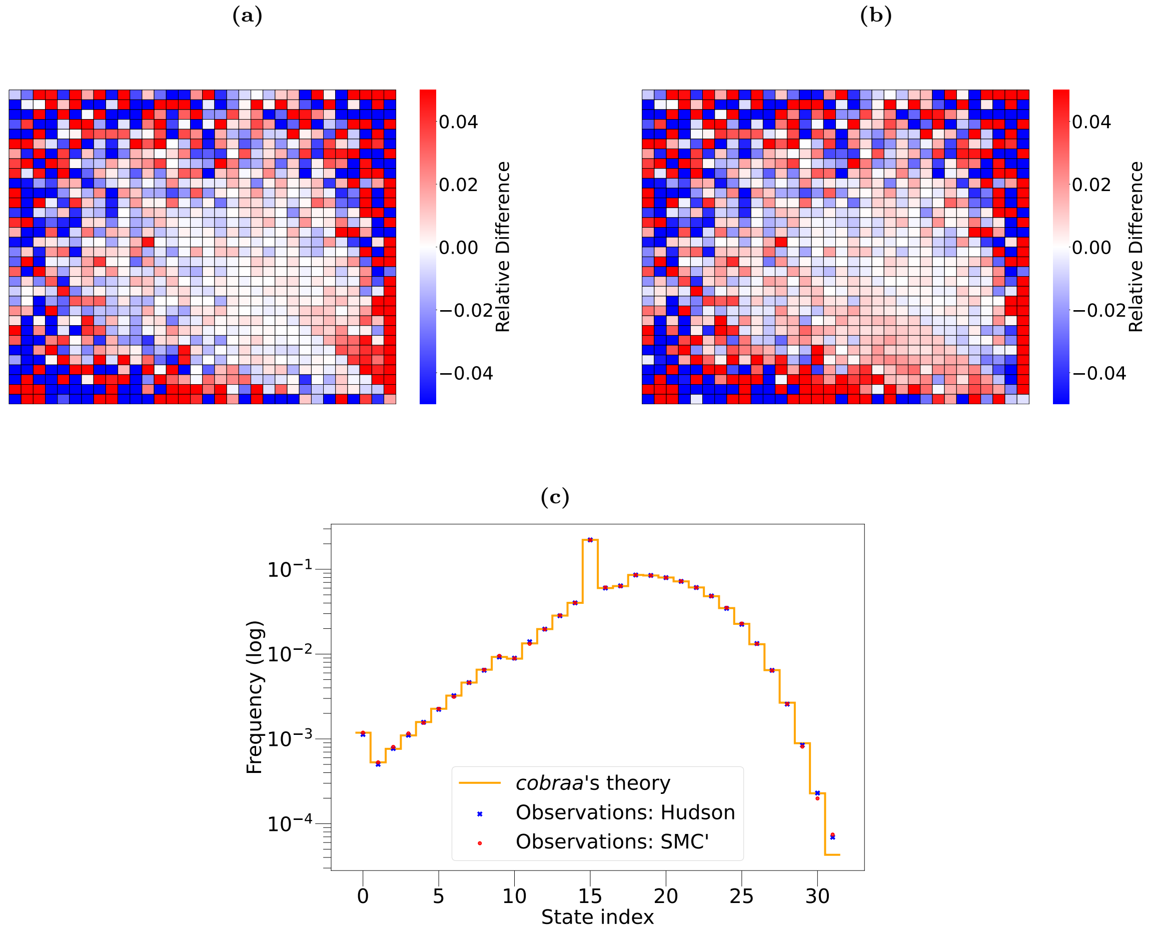

Supplementary Figure 18: Relative difference between the MLE and theoretical transition matrix $\xi = (\hat{Q}^{Hudson} - Q)/Q$. **(a)** Uses the expected coalescence time and **(b)** uses the midpoint of each discrete time window. **(c)** The (log) probability of transition probabilities given $\beta = 15$ (row 15 of $\xi = (\hat{Q}^{Hudson} - Q^{pulse})/Q^{pulse}$), according to a simulation with the Hudson (blue star) and SMC (red point) model, with *cobraa*'s theoretical prediction (orange line) given by Q^{pulse}.

This possibly explains the variance between the Hudson and SMC model. Qualitatively, the theoretical rate is extremely similar to both simulated distributions, except in ancient states which are again explainable by the inaccuracy of $\langle t_\beta \rangle$ for increasingly large time inteval boundaries.

Finally, we can perform a statistical hypothesis test to see if there is a significant difference between the theory and simulations. Using a Chi-squared test for each row α, we compute the test statistic with

$$\chi^2_\alpha = \sum_{i=1}^{D} \frac{(\hat{A}_{\alpha i} - E_{\alpha i})^2}{E_{\alpha i}}$$

where $E_{\alpha i} = \sum_{j=1}^{D} \hat{A}_{\alpha j} Q_{\alpha i}$ and set the degrees of freedom to $D - 1$. We do not reject the null hypothesis that Q_α provides a suitably good fit to the simulated data, because none of the p-values for the Hudson or SMC model exceed a 5% level of significance.

Supplementary Fig. 17, 18, and the statistical hypothesis testing suggests that our theory of marginal transitions of the *pulse* migration model under the SMC is a very good approximation to Hudson's coalescent with recombination model.

2.4 Inference from *cobraa* on simulations

In the main text we showed that *cobraa* has power to infer the parameters of a structured model, and that it can be used to distinguish structured or unstructured models. In this section, we perform similar experiments but over a larger parameter space, and in particular we highlight some scenarios in which *cobraa*'s power is limited. We also discuss the algorithm getting stuck in local optima and how this can be overcome.

Using msprime [14], we simulated a series of structured models with $\gamma \in$ [0.05,0.1,0.2,0.3,0.4], $(T_1, T_2) \in$ [(70k,293k), (130k,540k), (130k,993k), (293k,1.49M), (293k,2.23M)], and $N_A(t) = 16,000$ for all t except from $t < T_1$ where there is a bottleneck and subsequent recovery. We set $N_B(t) = 16,000$ in the structured period. We set μ=1.25e-08 and r=1e-08 and simulated 10 replicates of 3Gb of sequence data. For each of these structured models, we also simulated a corresponding unstructured model with population size changes such that it has a matching coalescence rate profile. On each simulation, we ran *cobraa* and PSMC inference until convergence. In *cobraa*, we fixed T_1, T_2, and $N_B(t)$ at their simulated values, and experimented with different starting values for the admixture fraction in the EM algorithm, $\gamma_0 \in$ [0.05,0.2,0.4] (we emphasize that we use γ to denote the simulated admixture fraction and γ_0 to indicate *cobraa*'s first guess of the admixture fraction in the EM algorithm).

We show inference of $N_A(t)$ on structured models in Supplementary Fig. 19, where the black line is the simulated size of $N_A(t)$, the purple line is the inverse coalescence rate (ICR) of this simulation, the blue line is the PSMC inference of $N_A(t)$, the red line is the *cobraa* inference of $N_A(t)$ with $\gamma_0 = 0.2$, and the green, dashed lines indicate the simulated T_1 and T_2. The value of γ that *cobraa* inferred is shown in Supplementary Fig. 20, where the solid, horizontal line indicates the simulated γ in the structured model, the dashed, horizontal line indicates $\gamma = 0$ for the unstructured model, the different shapes represent different γ_0, the red data points indicate inference on a structured simulation, and the blue data points indicate inference on an unstructured simulation. The difference in log-likelihood for the structured model and the unstructured model, $\Delta_{\mathcal{L}} = \mathcal{L}_S - \mathcal{L}_U$, is shown in Supplementary Fig. 21, where again different shapes represent different γ_0, the red data points indicate inference on a structured simulation, and the blue data points indicate inference on an unstructured simulation.

We see that if T_1 and T_2 are recent and have small separation time (top two panels of Supplementary Fig. 19, 20, and 21) then *cobraa* does not perform overly well. In particular, the population size in the structured period is not well recovered (Supplementary Fig. 19), the inferred γ is inaccurate and unable to escape its starting value (Supplementary Fig. 20), and the structured model can not be clearly distinguished from the unstructured model (Supplementary Fig. 21). This is perhaps unsurprising as there is limited coalescence density this close to the present. Limited accuracy is likely an example of the EM algorithm getting stuck in local optima, as shown in Supplementary Fig. 22, where we see that γ_i is often unable to sufficiently navigate the likelihood surface and locate the simulated value. Converging in non global optima means that the unstructured model (PSMC) can achieve a greater likelihood than the structured model (*cobraa*), as

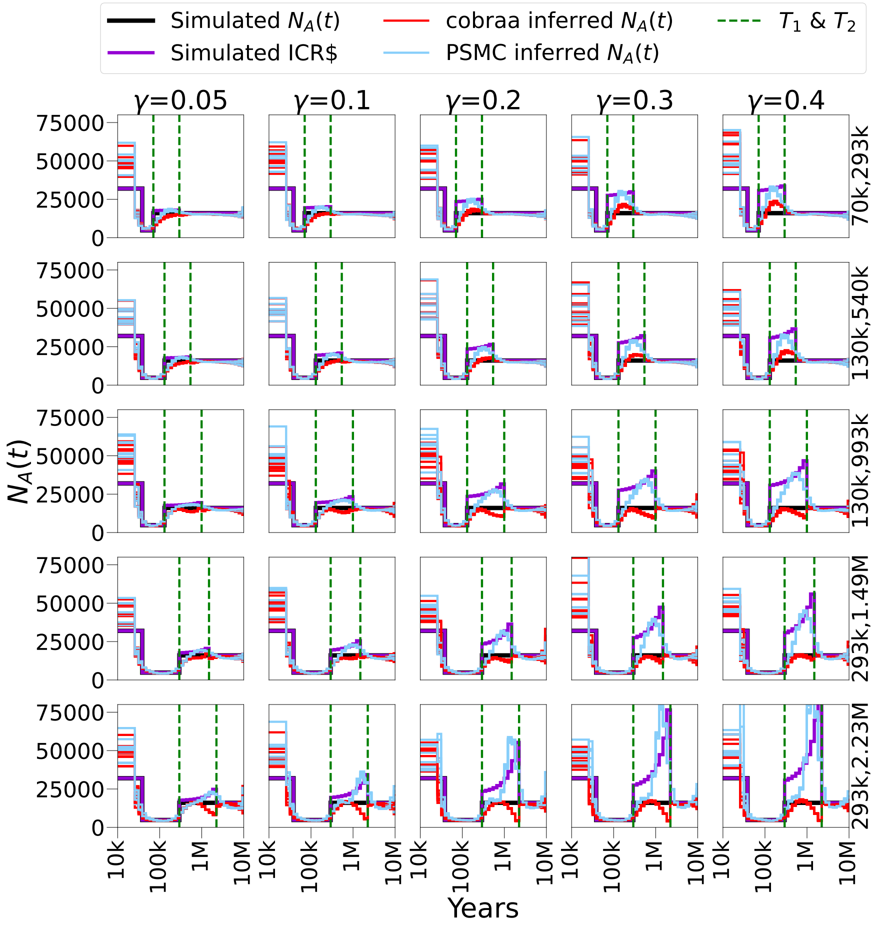

Supplementary Figure 19: Inference of $N_A(t)$ from PSMC and *cobraa* on a series of structured models. The simulated admixture fraction γ varies per column and is shown at the top of the figure. The simulated split/admixture times varies per row and is shown at the right of the figure. The black line is the simulated size of $N_A(t)$, the purple line is the inverse coalescence rate (ICR), the blue line is the PSMC inference of $N_A(t)$, the red line is the *cobraa* inference of $N_A(t)$ (with $\gamma_0 = 0.2$), and the green, dashed lines indicate the simulated T_1 and T_2. 10 replicates are shown.

seen in Supplementary Fig. 21, even though the former is nested in the latter (corresponding to $\gamma = 0$).

As T_1 and T_2 increase, however, the structured parameters are increasingly recoverable and can be clearly distinguished from unstructured models (bottom three panels panels of Supplementary Fig. 19, 20, 21 and 22). In particular, the simulated γ is consistently well recovered, even if it is as small as 0.05, irrespective of γ_0. We also see a clean separation in $\Delta_{\mathcal{L}}$, for all values of γ and γ_0. Finally, we highlight an important observation that there were no panmictic simulations for which the likelihood of the structured model exceeded the likelihood of the unstructured model.

2.4.1 Identifiability of structured model parameters

In the main text, we stipulated that the size of population B must be constant, N_B. The theory in the previous section allows time variance in the ghost's population size, $N_B(t)$, though we found significant

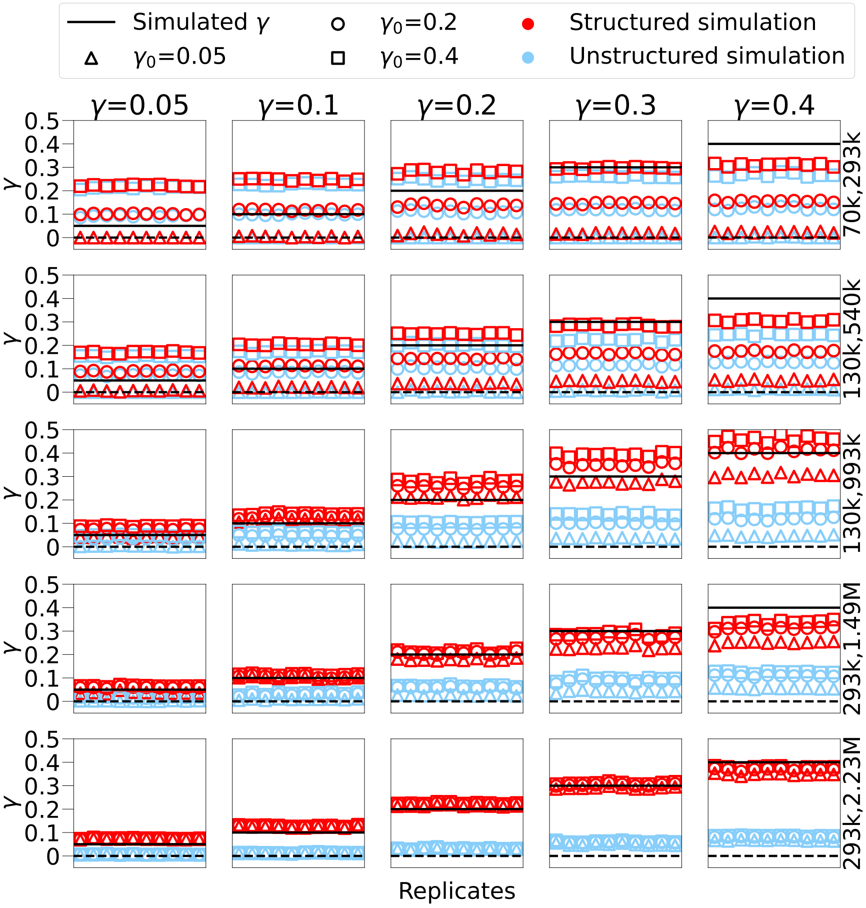

Supplementary Figure 20: Inference of γ from *cobraa* on a series of structured or unstructured models (the unstructured models have $\gamma = 0$ but a series of population size changes such that they have the same coalescence rate as the structured model). The simulated admixture fraction γ varies per column and is shown at the top of the figure. The simulated split/admixture times varies per row and is shown at the right of the figure. The solid, horizontal, line indicates the simulated γ in the structured model, the dashed, horizontal line indicates $\gamma = 0$ for the unstructured model, the different shapes represent different γ_0, the red data points indicate inference on a structured simulation, and the blue data points indicate inference on an unstructured simulation. The x-axis per subplot corresponds to each of the 10 replicates.

identifiability problems in distinguishing between $N_A(t)$ and $N_B(t)$, as discussed below.

2.4.1.1 EM analysis

We test how well the population size parameters and admixture fraction can be inferred by *cobraa*, provided that the split times are known. The ability of a coalescent HMM to perform accurate inference is dependent on the ratio of mutation rate to recombination rate μ/r. Initially, to test *cobraa* under a relatively easy scenario, we set $\mu/r = 10$ and simulated 10 replicates of 3Gb of a structured model with changes in $N_A(t)$ or $N_B(t)$, $T_1 = 250$ka, T_2=1Ma, and $\gamma = 0.4$. We run *cobraa* until convergence (defined as the change in log-likelihood being less than one, $\psi_{\mathcal{L}} < 1$, in subsequent iterations of the EM algorithm) on 10 replicates, and plot the inference in Supplementary Fig. 23. The simulated $N_A(t)$ and $N_B(t)$ are shown in black and gold, respectively, with the inferred $N_A(t)$ and $N_B(t)$ shown in red and blue, respectively. The simulated split and admixture times are indicated by the vertical, green, dashed lines. In Supplementary Fig. 23a population A

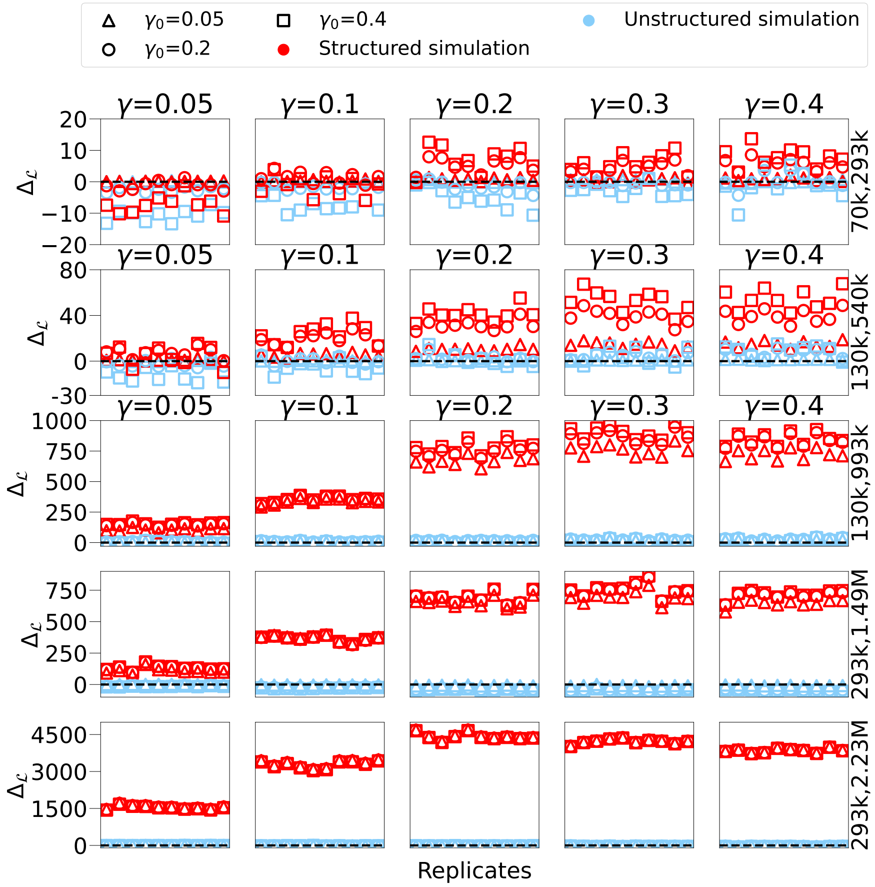

Supplementary Figure 21: The difference in log likelihoods on a series of structured or unstructured models (the unstructured models have $\gamma = 0$ but a series of population size changes such that they have the same coalescence rate as the structured model). The simulated admixture fraction γ varies per column and is shown at the top of the figure. The simulated split/admixture times varies per row and is shown at the right of the figure. The different shapes represent different γ_0, the red data points indicate inference on a structured simulation, and the blue data points indicate inference on an unstructured simulation. The x-axis per subplot corresponds to each of the 10 replicates.

doubles in size in the structured period whilst B stays constant, and vice-a-versa in Supplementary Fig. 23b.

We see that the split fraction is consistently estimated with reasonable accuracy, but the inferred $N_A(t)$ and $N_B(t)$ parameters are significantly biased. It seems that *cobraa* can not distinguish between changes in $N_A(t)$ or $N_B(t)$, and often one is overestimated at the expense of the other being underestimated. To examine this further, we investigated the likelihood surface.

2.4.1.2 Likelihood surface

We consider a structured model with constant size in A and B, an admixture fraction of 40%, and a separation time of ~800ka (with $D = 32$ time intervals, this corresponds to a split and admixture at the 18th and 10th discrete time interval, respectively). Using *msprime* we simulate under Hudson's model [15, 16] and record the sequence of marginal coalescence trees Z. We discretise this sequence of continuous coalescence times into the time windows used by *cobraa* (equation (8)), $\bar{Z} = \lfloor Z \rfloor_T$. Treating $\bar{Z}$ as a Markov chain, we can

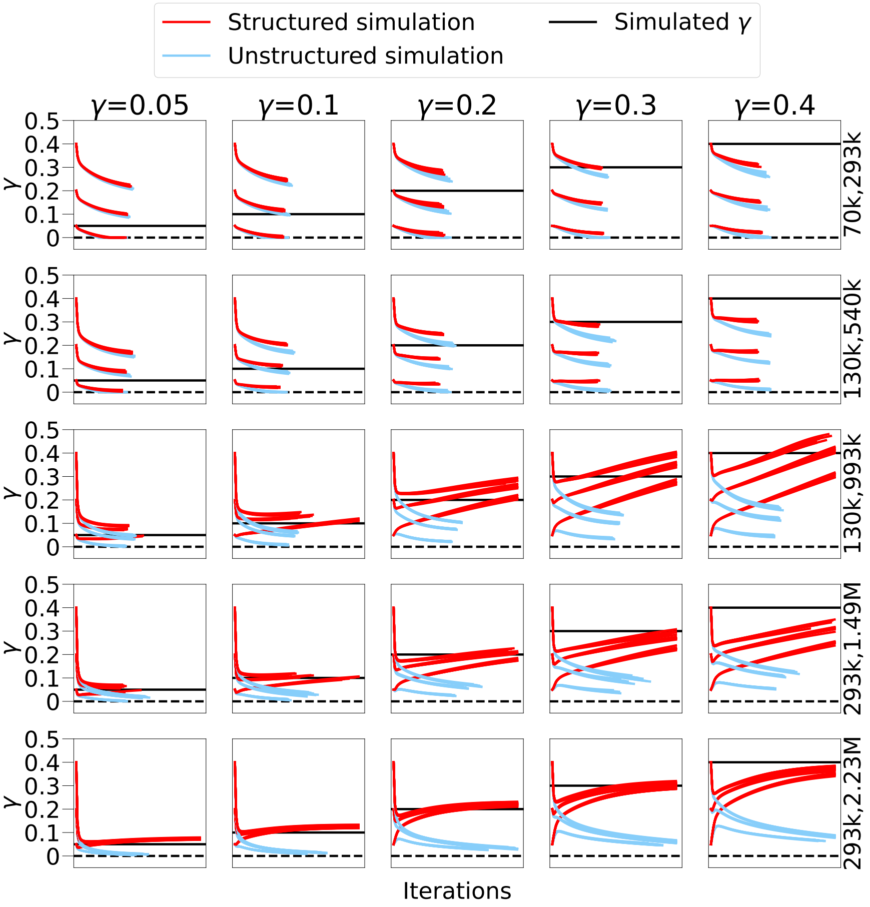

Supplementary Figure 22: The inferred admxiture fraction for a series of structured or unstructured models (the unstructured models have $\gamma = 0$ but a series of population size changes such that they have the same coalescence rate as the structured model). The simulated admixture fraction γ varies per column and is shown at the top of the figure. The simulated split/admixture times varies per row and is shown at the right of the figure. The red lines indicate inference on a structured simulation, and the blue lines indicate inference on an unstructured simulation. The x-axis per subplot corresponds to each iteration in the EM algorithm.

then calculate the log-likelihood of the sequence from *cobraa* under a particular set of parameters $\hat{\lambda}_A$, $\hat{\lambda}_B$, $\hat{\gamma}$, $\hat{T}_1$ and $\hat{T}_2$, by performing

$$P(\bar{Z}|\hat{\lambda}_A, \hat{\lambda}_B, \hat{\gamma}, \hat{T}_1, \hat{T}_2) = \sum_{i=1}^{L} log(Q(\bar{Z}_{i+1}|\bar{Z}_i)) \tag{66}$$

where Q is the *pulse* transition matrix (see section 2.3.1) defined by $\hat{\lambda}_A$, $\hat{\lambda}_B$, $\hat{\gamma}$, $\hat{T}_1$ and $\hat{T}_2$.

Using $S \in \{1, \ldots, D-1\}$ as the time index for T_1 and $E \in \{1, \ldots, D-1\}$ as the time index for T_2, with strictly $E > S$, then an unconstrained structured model has $D + (E - S) + 3$ parameters; D for λ_A, $(E - S)$ for λ_B, and one for each γ, T_1 and T_2. The resulting likelihood function of this unconstrained model therefore is of high dimensionality, making it computationally intractable to simulate under a wide range of values and not amenable to a simple two or three dimensional visualisation. However, as we are interested

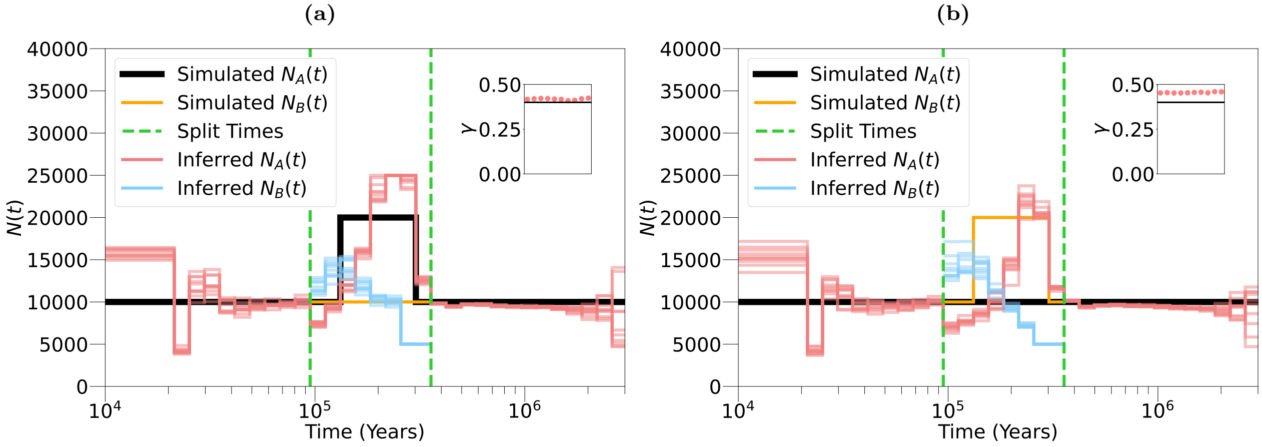

Supplementary Figure 23: Inference of population size changes for both populations A and B, and the admixture fraction. Simulated population sizes of population A and B are shown by the solid red and blue lines respectively. The inferred population sizes of A and B are shown by the light red and light blue lines respectively. The vertical, dashed green lines indicate the simulated split and admixture time of the model, and the inferred γ is detailed in the inset. Each plot shows a structured model with 40% admixture fraction and changes in (**(a)**) $N_A(t)$ or **(b)** $N_B(t)$ in the structured period.

in the unidentifiability of the effective size parameters for populations A and B within the structured period ($T_1 \leq t < T_2$), we can reduce the dimensionality to capture the essence of the problem by setting $\hat{\lambda}_{A_{10:18}}$ and $\hat{\lambda}_{B_{10:18}}$ each at one value. Outside the structured period, we fix the effective population size parameters of population A and the split/admixture times at their simulated value, $\hat{\lambda}_{A_{0:9}} = \hat{\lambda}_{A_{19:32}} = \mathbf{1}$, $\hat{T}_1 = 10$ and $\hat{T}_2 = 18$. We then let $\hat{\lambda}_{A_{10:18}}$, $\hat{\lambda}_{B_{10:18}}$ and $\hat{\gamma}$ vary over a grid of values and calculate the likelihood of the sequence under these parameters (equation (66)).

Three visualisations of the three-dimensional likelihood surface are shown in Supplementary Fig. 24. In Supplementary Fig. 24a, a plane of the surface is shown when $\hat{\gamma} = 0.4$ is fixed at its simulated value, to see how the likelihood changes as $\hat{\lambda}_{A_{10:18}}$ and $\hat{\lambda}_{B_{10:18}}$ vary. In Supplementary Fig. 24b and Supplementary Fig. 24c, $\hat{\lambda}_{B_{10:18}}$ and $\hat{\lambda}_{A_{10:18}}$ respectively are fixed at their simulated values, to get an idea of how the surface varies with $\hat{\gamma}$. In each, the green highlighted cell indicates the simulated value of the non-fixed parameters. Supplementary Fig. 24a illustrates that there is an extended region in the space of $(\hat{\lambda}_{A_{10:18}}, \hat{\lambda}_{B_{10:18}})$ values that achieves a likelihood comparable to the simulated value, seemingly by trading off changes in the two variables.

Generally speaking, if and only if λ_A and λ_B are equal everywhere in the structured period, then with respect to γ the model is symmetric about $\gamma = 0.5$. This is because population A and B are exchangeable in this period if we set $\gamma_{B,A} = 1 - \gamma_{A,B}$. We see this from the fact that the surface in Supplementary Fig. 24c is a vertical mirror of 24b. Furthermore (less obvious from the images) both Supplementary Fig. 24b and 24c have dual likelihood peaks at $\hat{\gamma} = 0.4$ and $\hat{\gamma} = 0.6$. From 24b, it appears that at the simulated split fraction $\hat{\gamma} = 0.4$, the likelihood function is sensitive to the value of $\hat{\lambda}_{A_{10:18}}$, due to the sharp drops seen around its simulated value, though at the symmetric split fraction $\hat{\gamma} = 0.6$ it appears to be less so, as at this axis the likelihood decreases at a slower rate. Note that the reverse is true in 24c: at the simulated split fraction $\hat{\gamma} = 0.4$, the likelihood function is not as sensitive to $\hat{\lambda}_{B_{10:18}}$ as at the symmetric split fraction $\hat{\gamma} = 0.6$.

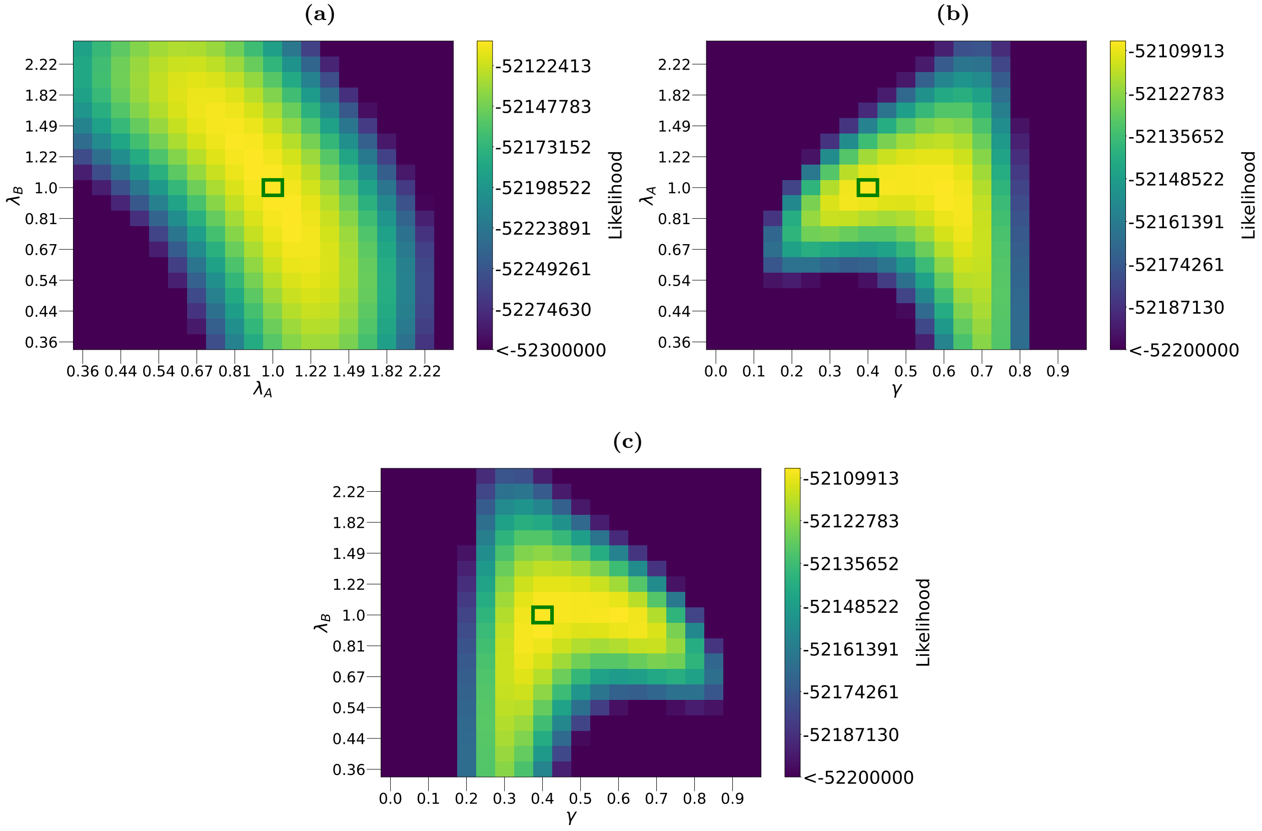

Supplementary Figure 24: Three contour plots from the likelihood surface for a structured model. The colourbar indicates the log-likelihood, and the green cell indicates the simulated values. Shown in **(a)** is variation between ($\hat{\lambda}_{A_{10:18}}$,$\hat{\lambda}_{B_{10:18}}$) with $\hat{\gamma}$ fixed at its simulated value; in **(b)** is variation between ($\hat{\gamma}$,$\hat{\lambda}_{A_{10:18}}$) with $\hat{\lambda}_{B_{10:18}}$ fixed at its simulated value; in **(c)** is variation between ($\hat{\gamma}$,$\hat{\lambda}_{B_{10:18}}$) with $\hat{\lambda}_{A_{10:18}}$ fixed at its simulated value.

We note that this analysis was performed on the sequence of known marginal coalescence times $\bar{Z}$ which we treat as a Markov chain, from which it is straightforward to calculate the likelihood (equation (66)). In practise, we do not know the coalescence times $\bar{Z}$ along the genome, so with a sequence of observations X we formulate a HMM and calculate the likelihood of the data $P(X|\hat{\lambda}_A, \hat{\lambda}_B, \hat{\gamma}, \hat{T}_S, \hat{T}_E)$ with dynamic programming. Only knowing the observed sequence X will necessarily flatten the peaks of the likelihood surface, due to the uncertainty of $\bar{Z}$ which must be probabilistically inferred, making optimising over the parameter space even harder in reality. We also emphasise that this experiment was performed with the effective population size changes for population A and B each set to one value in the structured period. In reality, we would hope for more freedom, but this stipulation allowed us to demonstrate the difficulty in navigating the parameter space even under simplified conditions.

In summary, Supplementary Fig. 24 demonstrates that even with a simple evolutionary history and constraining the effective population size parameters to an equal value in the structured period, there appears to be numerous regions of high likelihood that do not correspond to the simulated parameters. This indicates that using the EM algorithm may be insufficient in ascertaining the maximum likelihood parameters, due to the sensitivity of the optimisation's starting guess and the difficulty in navigating the parameter space.

This justifies our requirement that population B is of constant size.

2.5 *cobraa-path*'s HMM

The hidden states of *cobraa* are coalescence time windows, meaning that when we calculate the probability of transitioning we had to include all the possible paths that a pair of lineages could have taken through the *pulse* model. This is unnatural, and condenses a lot of information into one hidden state. Instead, we can expand the HMM of *cobraa* such that the hidden state represents not just the coalescence time, but also the ancestral path taken through the two populations before the two sampled lineages coalesce. If the coalescence time occurs more recently than the admixture event, then the only possibility is that both lineages coalesced in population A, which we denote as AA. If the coalescence occurred in the structured period, then there are two possible paths, AA and BB. If the coalescence occurred more anciently than the population divergence, then there are three possible paths, AA, BB and AB. An illustration is given in Extended Data Fig. 4.

We call this new HMM *cobraa-path*. The hidden states are then a tuple (t, c) where $c \in (AA, BB, AB)$ is the ancestral path taken by the two lineages which coalesce at time t. The emission probabilities are similar to *cobraa*'s, though given t are repeated across different values of c. For example given a coalescence of t that is more ancient than population divergence, the probability of observing a mutation is the same for each $c \in AA, BB, AB$. We also note that if $\gamma = 0$ then *cobraa-path* reduces to standard PSMC.

The transition probabilities of *cobraa-path* follow from *cobraa*. Let $Q_X(\alpha, j|\beta, k) = P_X(z_{i+1} = \tau_\alpha, c_{i+1} = j|z_i = T_\beta, c_i = k)$. Using Case 3 as an illustrative example, we can decompose equation (31) into its constituent parts to rapidly obtain:

$$Q_3^{pulse}(\alpha, AA|\beta, AA) = \frac{\lambda_{A_\alpha} P(R|\langle t_\beta \rangle)}{\langle t_\beta \rangle} \Big(K_A^2(\tau_0, T_1)(1-\gamma)L_A(T_1, \tau_\alpha)^2 J_A^2(\tau_\alpha) + K_A^2(T_1, \tau_\alpha)J_A^2(\tau_\alpha) + H_A^2(\tau_\alpha) \Big) \tag{67}$$

$$Q_3^{pulse}(\alpha, BB|\beta, AA) = 0 \tag{68}$$

$$Q_3^{pulse}(\alpha, BB|\beta, BB) = \frac{\lambda_{B_\alpha} P(R|\langle t_\beta \rangle)}{\langle t_\beta \rangle} \Big(K_A^2(\tau_0, T_1)\gamma L_B(T_1, \tau_\alpha)^2 J_B^2(\tau_\alpha) + K_B^2(T_1, \tau_\alpha)J_B^2(\tau_\alpha) + H_B^2(\tau_\alpha) \Big) \tag{69}$$

$$Q_3^{pulse}(\alpha, AA|\beta, BB) = 0 \tag{70}$$

$$Q_3^{pulse}(\alpha, AA|\beta, AB) = \frac{\lambda_{A_\alpha} P(R|\langle t_\beta \rangle)}{2\langle t_\beta \rangle} K_A^2(\tau_0, T_1)\Big((1-\gamma)L_A(T_1, \tau_\alpha)J_A^1(\tau_\alpha)\Big) \tag{71}$$

$$Q_3^{pulse}(\alpha, BB|\beta, AB) = \frac{\lambda_{B_\alpha} P(R|\langle t_\beta \rangle)}{2\langle t_\beta \rangle} K_A^2(\tau_0, T_1)\Big(\gamma L_B(T_1, \tau_\alpha)J_B^1(\tau_\alpha)\Big). \tag{72}$$

All the other cases follow similarly.

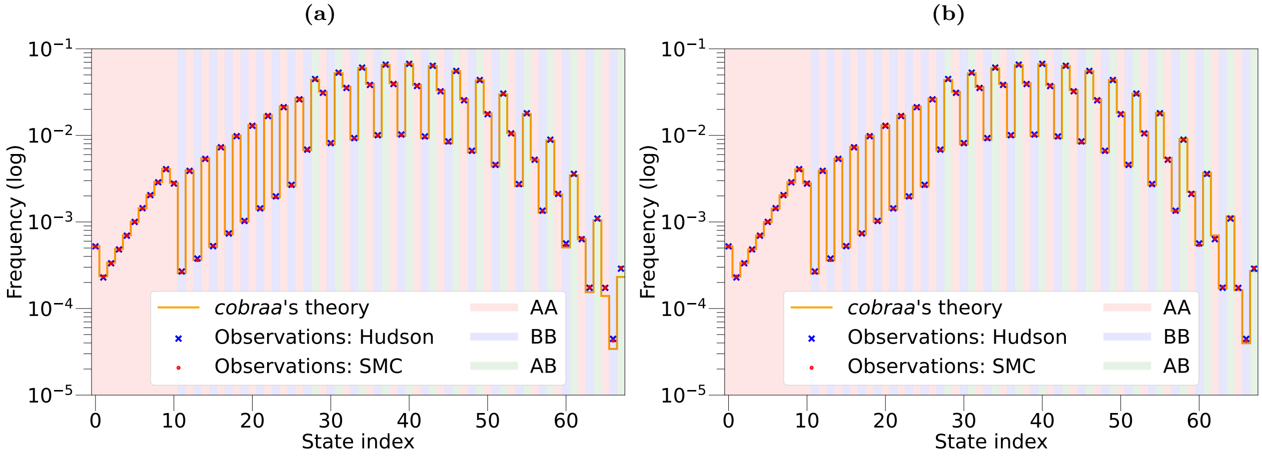

Supplementary Figure 25: The (log) proportion of time spent in each state, according to a simulation with the Hudson (blue star) and SMC (red point) model, with the theoretical proportion from *cobraa-path* (orange line). **(a)** Using the expected coalescence time in each interval **(b)** Using the midpoint of each interval. The shading represents which ancestral path the hidden state corresponds to, with red being AA, blue being BB and green being AB.

2.5.1 Demonstration of correctness

We simulate marginal transition data from msprime, and record the appropriate lineage path and coalescence time information at each position, using both the Hudson and SMC model. We then compare the observed proportion of time spent in each state to *cobraa-path*'s theory in Supplementary Fig. 25, which indicates the theory is a very good fit to the data.

References

[1] Olivier Mazet, Willy Rodríguez, and Lounès Chikhi. Demographic inference using genetic data from a single individual: Separating population size variation from population structure. *Theoretical Population Biology*, 104:46–58, 2015.

[2] Olivier Mazet, Willy Rodríguez, Simona Grusea, Simon Boitard, and Lounès Chikhi. On the importance of being structured: instantaneous coalescence rates and human evolution—lessons for ancestral population size inference? *Heredity*, 116(4):362–371, 2016.

[3] Trevor Cousins, Daniel Tabin, Nick Patterson, David Reich, and Arun Durvasula. Accurate inference of population history in the presence of background selection. *Preprint at biorxiv.org/content/10.1101/2024.01.18.576291v1*, pages 2024–01, 2024.

[4] Paul D Thomas, Dustin Ebert, Anushya Muruganujan, Tremayne Mushayahama, Laurent-Philippe Albou, and Huaiyu Mi. Panther: Making genome-scale phylogenetics accessible to all. *Protein Science*, 31(1):8–22, 2022.

[5] Gilean AT McVean and Niall J Cardin. Approximating the coalescent with recombination. *Philosophical Transactions of the Royal Society B: Biological Sciences*, 360(1459):1387–1393, 2005.

[6] Carsten Wiuf and Jotun Hein. Recombination as a point process along sequences. *Theoretical Population Biology*, 55(3):248–259, 1999.

[7] Paul Marjoram and Jeff D Wall. Fast "coalescent" simulation. *BMC Genetics*, 7(1):1–9, 2006.

[8] Peter R Wilton, Shai Carmi, and Asger Hobolth. The SMC' is a highly accurate approximation to the ancestral recombination graph. *Genetics*, 200(1):343–355, 2015.

[9] Heng Li and Richard Durbin. Inference of human population history from individual whole-genome sequences. *Nature*, 475(7357):493–496, 2011.

[10] Ke Wang, Iain Mathieson, Jared O'Connell, and Stephan Schiffels. Tracking human population structure through time from whole genome sequences. *PLoS Genetics*, 16(3):e1008552, 2020.

[11] Vladimir Shchur, Débora YC Brandt, Anna Ilina, and Rasmus Nielsen. Estimating population split times and migration rates from historical effective population sizes. *Preprint at biorxiv.org/content/10.1101/2022.06.17.496540v1*, 2022.

[12] Anna-Sapfo Malaspinas, Michael C Westaway, Craig Muller, Vitor C Sousa, Oscar Lao, Isabel Alves, Anders Bergström, Georgios Athanasiadis, Jade Y Cheng, Jacob E Crawford, et al. A genomic history of Aboriginal Australia. *Nature*, 538(7624):207–214, 2016.

[13] Stephan Schiffels and Richard Durbin. Inferring human population size and separation history from multiple genome sequences. *Nature Genetics*, 46(8):919–925, 2014.

[14] Jerome Kelleher, Alison M Etheridge, and Gilean McVean. Efficient coalescent simulation and genealogical analysis for large sample sizes. *PLoS Computational Biology*, 12(5):e1004842, 2016.

[15] Richard R Hudson. Properties of a neutral allele model with intragenic recombination. *Theoretical Population Biology*, 23(2):183–201, 1983.

[16] Richard R Hudson et al. Gene genealogies and the coalescent process. *Oxford Surveys in Evolutionary Biology*, 7(1):44, 1990.
